# NaCl improves reproduction by enhancing starch accumulation in the ovules of the euhalophyte *Suaeda salsa*

**DOI:** 10.1186/s12870-020-02468-3

**Published:** 2020-06-08

**Authors:** Jianrong Guo, Ming Du, Chaoxia Lu, Baoshan Wang

**Affiliations:** Shandong Provincial Key Laboratory of Plant Stress, College of Life Sciences, Shandong Normal University, Ji’nan, Shandong 250014 People’s Republic of China

**Keywords:** Carbohydrate metabolism, Genes, NaCl, *Suaeda salsa* L., Reproductivity

## Abstract

**Background:**

Halophytes show optimal reproduction under high-salinity conditions. However, the role of NaCl in reproduction and its possible mechanisms in the euhalophyte *Suaeda salsa* remain to be elucidated.

**Results:**

We performed transcript profiling of *S. salsa* flowers and measured starch accumulation in ovules, sugar contents in flowers, and photosynthetic parameters in the leaves of plants supplied with 0 and 200 mM NaCl. Starch accumulation in ovules, sugar contents in flowers and ovules, and net photosynthetic rate and photochemical efficiency in leaves were significantly higher in NaCl-treated plants vs. the control. We identified 14,348 differentially expressed genes in flowers of NaCl-treated vs. control plants. Many of these genes were predicted to be associated with photosynthesis, carbon utilization, and sugar and starch metabolism. These genes are crucial for maintaining photosystem structure, regulating electron transport, and improving photosynthetic efficiency in NaCl-treated plants. In addition, genes encoding fructokinase and sucrose phosphate synthase were upregulated in flowers of NaCl-treated plants.

**Conclusions:**

The higher starch and sugar contents in the ovules and flowers of *S. salsa* in response to NaCl treatment are likely due to the upregulation of genes involved in photosynthesis and carbohydrate metabolism, which increase photosynthetic efficiency and accumulation of photosynthetic products under these conditions.

## Background

Saline soil severely reduces crop production worldwide, especially in irrigated agricultural lands [1, 2]. Halophytes survive and reproduce in saline environments with salt concentrations of ≥200 mM [3] due to changes in plant physiology and biochemistry [1, 4]. Osmotic adjustment is the first process that occurs in plants experiencing saline environments. Plants can achieve osmotic balance by accumulating inorganic ions [5, 6] and organic solutes (proline, glycine betaine, and soluble sugars) [7–9]. Both the carbon and energy required for organic biosynthesis are derived from soluble sugars, which are crucial players in abiotic stress tolerance in plants [10]. Therefore, carbohydrate metabolism can be used as a physiological indicator to evaluate salt tolerance. During vegetative growth, the survival of euhalophytes is mainly attributable to the exclusion of Na^+^ and Cl^−^ and/or their sequestration into vacuoles and the maintenance of ionic homeostasis to avoid ionic toxicity in younger leaves [11–15].

Environmental stresses negatively affect plant productivity. For example, cold stress during the reproductive phase may disturb the structure and function of reproductive organs, thus leading to reduced or failed seed or fruit set [16]. High temperature markedly reduces crop yields by damaging male and female tissues and altering physiological processes that are highly sensitive during reproductive development [17]. Drought stress during the reproductive phase may induce pollen sterility in rice by inhibiting starch accumulation in pollen, as well as disturbing sugar and starch metabolism [18]. Poor seed set occurs under terminal drought stress due to inhibition of photosystem II (PSII) function [19] and a reduction in the net photosynthetic rate [20].

Although reproductive development is even more sensitive to salinity than vegetative development in crops [21–24], the molecular mechanisms underlying the effects of salinity stress on plant reproduction remain to be elucidated. The reduced plant yields induced by salinity are attributed to factors such as salt accumulation, potassium deficiency, restricted water flow, hormonal imbalance, and reduced carbon supply due to low photosynthetic efficiency [1, 25–27]. Halophytes show maximum growth during the vegetative and reproductive phases when high NaCl concentrations are present in the growth medium [3, 28, 29], even at levels that kill non-halophytes such as maize, wheat, and rice. The presence of NaCl stimulates flowering but reduces seed number in the halophyte *Plantago crassifolia* [30]. However, how halophytes respond to saline environments during the reproductive phase, especially in the molecular aspects of photosynthesis and carbohydrate metabolism, remains to be elucidated.

Photosynthesis is a fundamental process for plant growth and development that is affected by abiotic stresses such as drought, heat, and salinity [31]. For example, water deficiency decreases photosynthetic efficiency in *Jatropha curcas* leaves, which is accompanied by reduced sugar content [32]. In general, salinity decreases photosynthetic efficiency and inhibits the growth of non-halophytes, including major crops. For example, salinity reduces plant growth in rice by decreasing chlorophyll contents and disturbing the photosynthetic process [33]. However, the total soluble sugar contents and net photosynthesis are enhanced under salt stress in a salt-tolerant vs. salt-sensitive rice genotype, since carbohydrates provide energy resources to help salt-tolerant plants adapt to salt stress (13.25 deci siemens (dS) m^− 1^) [34]. By contrast, we previously demonstrated that the net photosynthesis rate significantly increased in the euhalophyte *Suaeda salsa* in response to 200 NaCl treatment [35–38].

*Suaeda salsa* L. is an edible euhalophyte found in northern China that grows well in natural saline soil environments. The optimal condition for both vegetative and reproductive growth of *S. salsa* is 200 mM NaCl in the growth medium [28, 38–40]. *S. salsa* is a promising model system for unraveling the salt tolerance mechanisms of euhalophytes [41]. During the vegetative growth phase, *S. salsa* adapts to saline environments by efficiently maintaining ionic balance [35, 42] and via active oxygen scavenging [14, 43, 44]. The addition of 200 mM NaCl to the growth medium markedly improves flower number, seed number, and seed production in *S. salsa* [28]. However, the relationship between carbohydrate metabolism and reproductive development in *S. salsa* under high-NaCl conditions is unknown, particularly with regard to the expression of genes involved in photosynthesis.

Here, we investigated how high salinity enhances reproductive development in *S. salsa* plants subjected to 200 mM NaCl treatment. We analyzed the transcriptomes of *S. salsa* flowers from control and NaCl-treated plants via high-throughput Illumina RNA sequencing (RNA-seq) and measured photosynthetic parameters in leaves, as well as carbohydrate indicators. And to investigate whether the genes involving in photosynthesis play important roles in the enhancement of halophyte *S. salsa* reproductive processes under high-salinity conditions, particularly photosynthesis in leaves and the translocation, accumulation, and utilization of photoassimilates in flowers.

## Results

### Seed size is enhanced in NaCl-treated *S. salsa*

We examined fruit development in control vs. NaCl-treated *S. salsa* plants during the reproductive period. Black seeds matured earlier than brown seeds on the same branchlet. Both black and brown seeds from NaCl-treated plants were significantly larger (*P* < 0.05) than those from control plants (Additional file 1: Figure S1). Additionally, the other parameters such as seed number and seed weight per plant, and the mean individual seed mass were also significantly enhanced as reported in our previous study [28].

### NaCl improves starch accumulation in *S. salsa* ovules

**Fig. 1:**
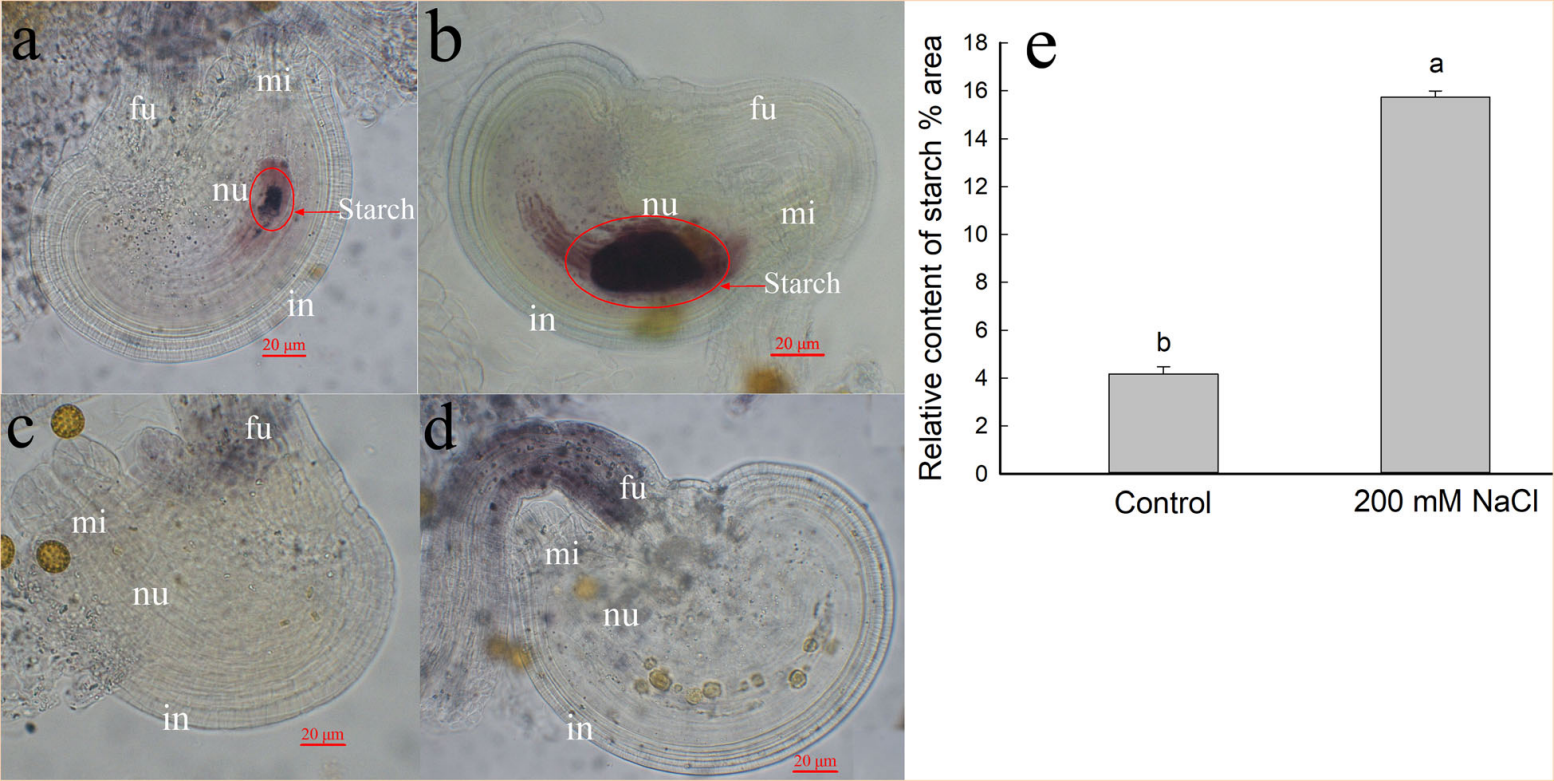
Lugol’s staining of ovules before and after fertilization from *S. salsa* plants grown in sand in medium containing 0 (**a** and **c**) and 200 mM NaCl (**b** and **d**) and the relative starch content in the ovule of *S. salsa* before fertilization (**e**). Ovules of plants under control (**a**) and 200 mM NaCl treatment (**b**) before fertilization, ovules of plants under control (**c**) and 200 mM NaCl treatment (**d**) after fertilization. mi, micropyle; fu, funiculus; nu, nucellus; in, integument

We measured starch accumulation in the embryo sacs of control and 200 mM NaCl-treated *S. salsa* plants (Fig. 1). Before fertilization (108 days after sowing (DAS)), starch levels were significantly higher (*P* < 0.05) in the ovules of NaCl-treated plants than in control plants (Fig. 1a, b). After fertilization (110 DAS), the accumulated starch is used for embryo and endosperm development [45]. Therefore, no starch accumulation was detected upon either control or 200 mM NaCl treatment after fertilization (Fig. 1c, d). However, the funiculi of the ovules of NaCl-treated plants accumulated more starch than those of the controls. The high levels of starch should provide enough raw materials for embryo development, perhaps explaining why seeds were larger in plants treated with 200 mM NaCl than in control plants.

### NaCl treatment increases total soluble sugar levels in leaves, stems, and flowers during early reproductive growth

**Fig. 2:**
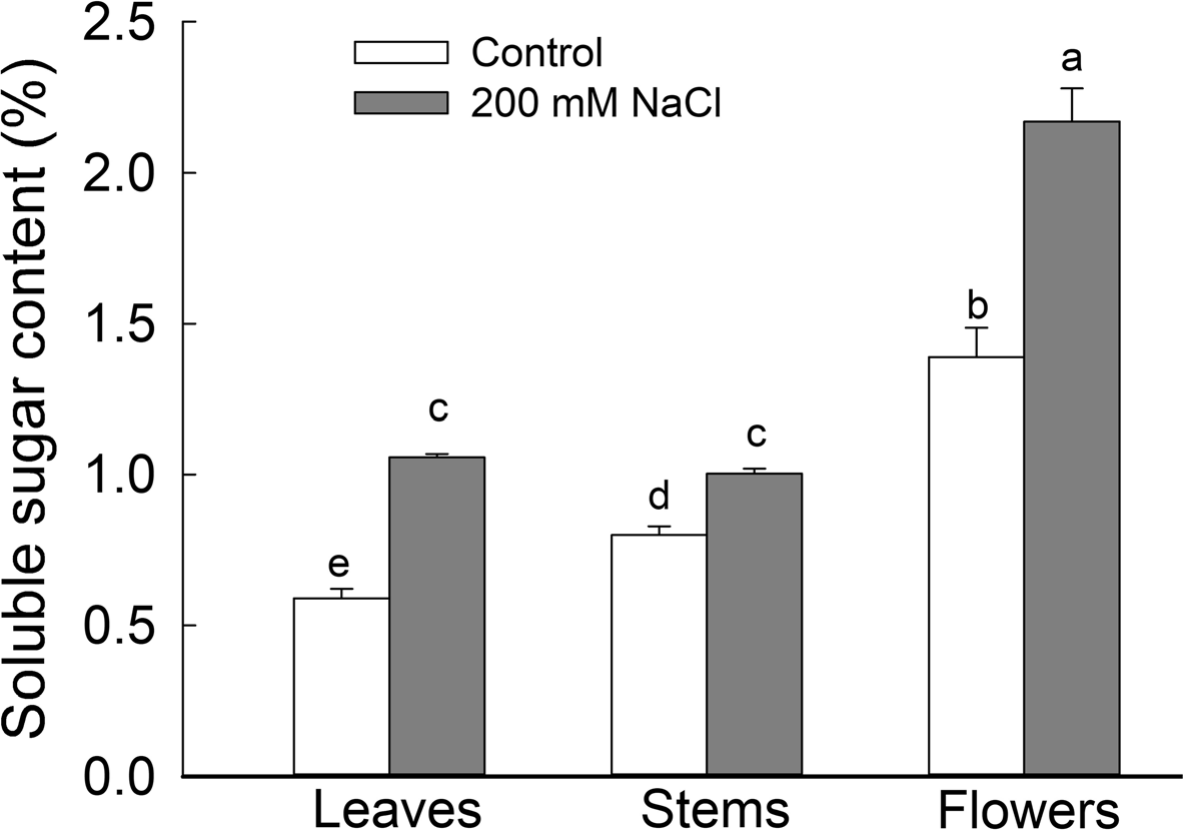
Total soluble sugar (TSS) contents in leaves, stems, and flowers of *S. salsa* plants grown in sand in medium containing 0 and 200 mM NaCl at the flowering stage (105 DAS). Values are presented as the means ± SD of four replicates. Different letters in the figure indicate a significant difference at *P* < 0.05 according to Duncan’s test

We measured total soluble sugar (TSS) contents in NaCl-treated and control *S. salsa* plants at the beginning of the reproductive growth phase (105 DAS). TSS contents were significantly higher (*P* < 0.05) in the leaves, stems, and flowers of 200 mM NaCl-treated plants compared to the controls, with levels 1.79-, 1.25-, and 1.56-fold those of the control, respectively. TSS levels were particularly elevated in the floral organs of plants treated with 200 mM NaCl, with levels 2.05- and 2.16-fold of those in leaves and stems, respectively (Fig. 2).

### NaCl increases the net photosynthesis rate in *S. salsa* leaves

**Fig. 3:**
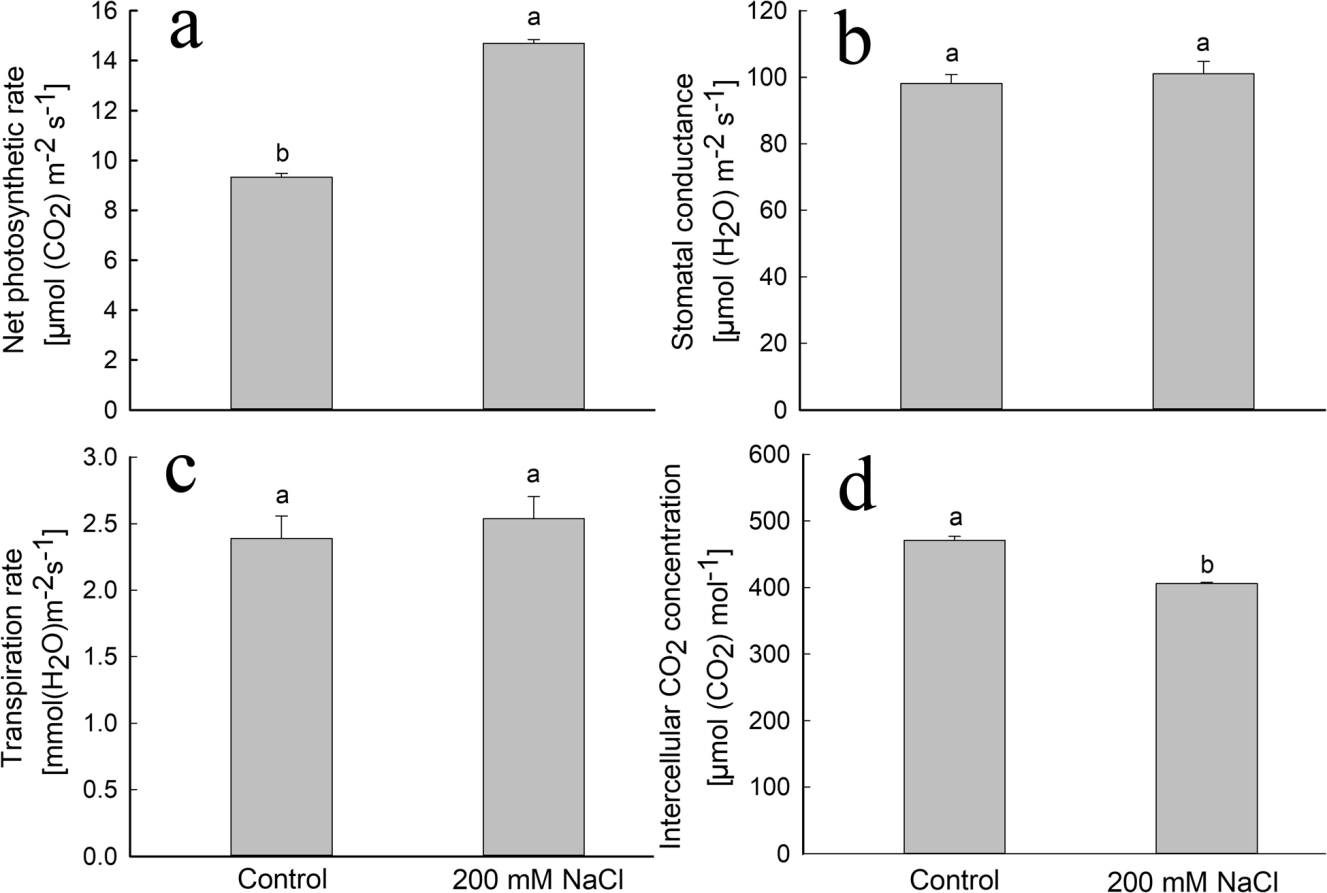
Net photosynthetic rate (*P*_n_) (**a**), stomatal conductance (*g*_s_) (**b**), transpiration rate (**c**), and intercellular CO_2_ concentration (**d**) of *S. salsa* plants grown in sand in medium containing 0 and 200 mM NaCl at the beginning of the reproductive growth stage (103 DAS). Values are presented as the means ± SD of 15 replicates. Different letters in the figure indicate a significant difference at *P* < 0.05 according to Duncan’s test

Starch and soluble sugars in plants are directly derived from photosynthesis. During the beginning of the reproductive growth period (103 DAS), the net photosynthetic rate was significantly (57.4%) higher (*P* < 0.05) in the leaves of *S. salsa* plants treated with NaCl compared to the control (Fig. 3a). However, we detected no significant difference in the transpiration rate or stomatal conductance between control and 200 mM NaCl-treated plants (Fig. 3b, c). By contrast, the intercellular CO_2_ concentration was 13.8% lower in NaCl-treated plants than in control plants (Fig. 3d).

### NaCl treatment increases chlorophyll contents in *S. salsa* leaves

**Fig. 4:**
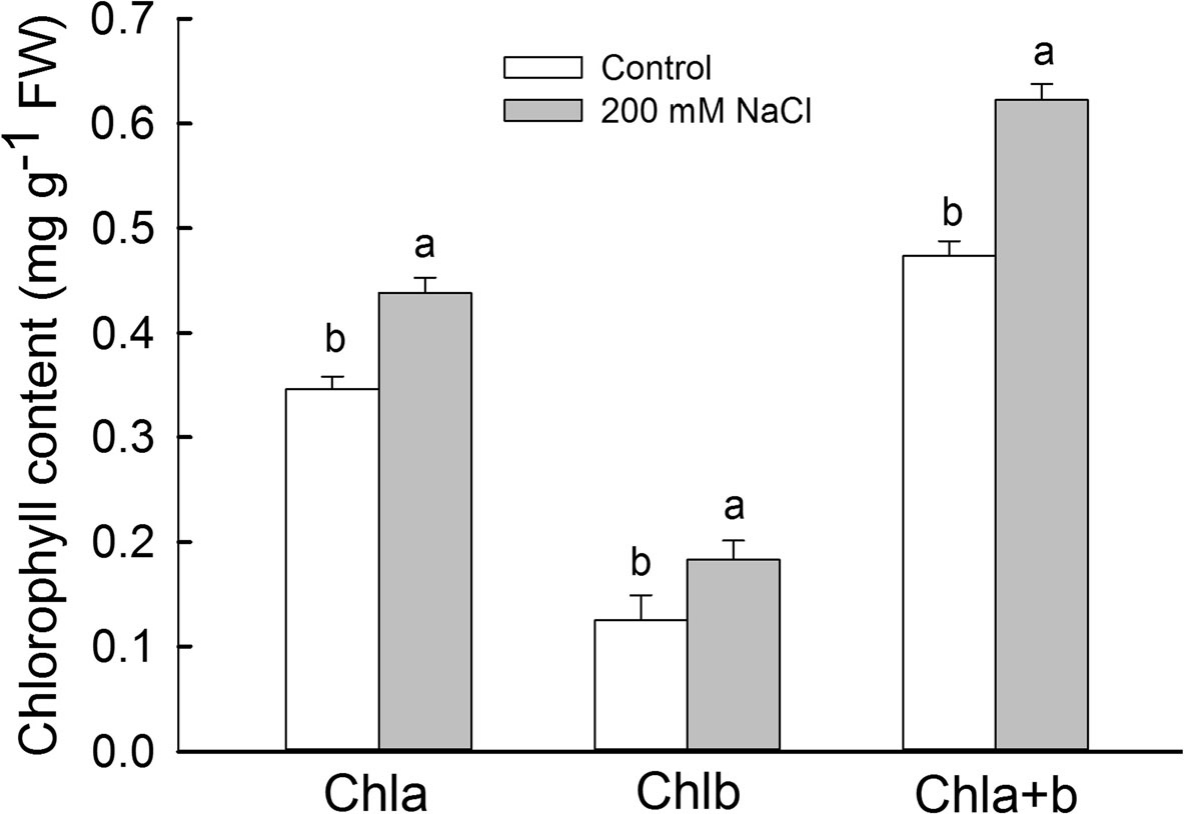
Chlorophyll contents in the leaves of *S. salsa* plants grown in sand in medium containing 0 and 200 mM NaCl at the beginning of the reproductive growth stage (103 DAS). Values are presented as the means ± SD of five replicates. Different letters in one group indicate a significant difference at *P* < 0.05 according to Duncan’s test

The efficiency of photosynthesis is related to chlorophyll contents in leaves. We therefore measured chlorophyll contents in the leaves of control and NaCl-treated *S. salsa* plants at 103 DAS. Chlorophyll contents were significantly higher (*P* < 0.05) in the leaves of NaCl-treated vs. control plants, including chlorophyll a, chlorophyll b, and total chlorophyll contents, with increases of 26.6, 46.4, and 31.5%, respectively (Fig. 4). A similar pattern was detected for chlorophyll contents in the flowers of *S. salsa* plants treated with NaCl (Additional file 2: Figure S2). During early flower development, the petals were green and contained chloroplasts (Additional file 3: Figure S3), indicating that *S. salsa* flowers can undergo photosynthesis during early development.

### NaCl treatment enhances photosynthetic oxygen evolution in *S. salsa* petals

Based on the existence of chloroplasts and chlorophyll in the petals of *S. salsa* flowers at early stage, the photosynthetic oxygen evolution in petals was determined. It was showed that the oxygen release capacity in petals of *S. salsa* treated with NaCl was significantly higher (*P* < 0.05) than that in the control plants (Additional file 4: Figure S4), in spite of that the photosynthetic capacity was much lower than that in leaves (Fig. 3a).

### NaCl treatment enhances photochemical efficiency of PSII in *S. salsa* leaves

**Fig. 5:**
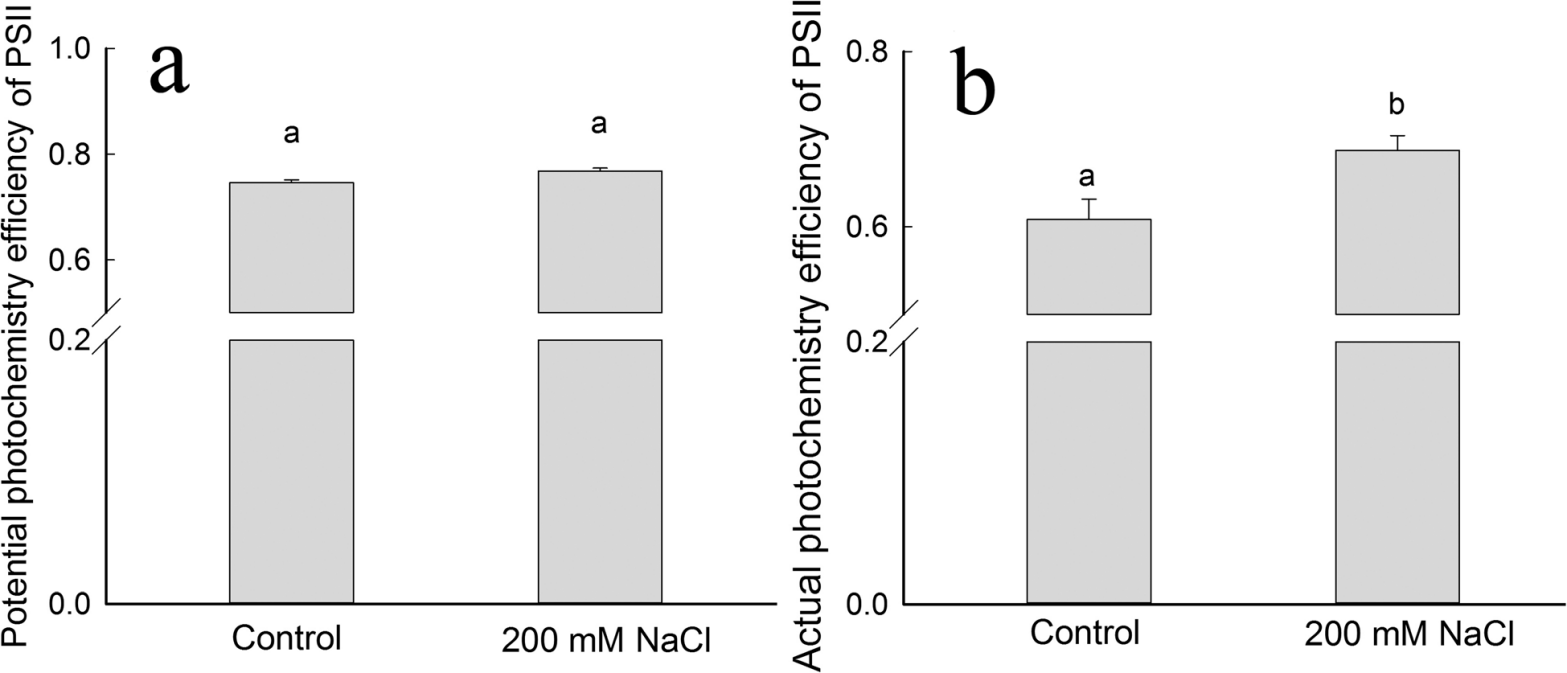
Maximal quantum yield of PSII (*F*_v_/*F*_m_) (a) and effective quantum yield of PSII (Φ_PSII_) (**b**) of *S. salsa* plants grown in sand in medium containing 0 and 200 mM NaCl at the beginning of the reproductive growth stage (103 DAS). Values are presented as the means ± SD of five replicates. Different letters indicate a significant difference at *P* < 0.05 according to Duncan’s test

Both the potential photochemistry efficiency (*F*_v_/*F*_m_) and actual photochemistry efficiency (Φ_PSII_) of PSII were significantly higher (*P* < 0.05) in the leaves of NaCl-treated plants vs. the control (Fig. 5). However, there was no significant difference in *F*_v_/*F*_m_, an indicator of the maximum quantum yield of PSII in dark-adapted leaves, between the two treatment groups (Fig. 5a). By contrast, Φ_PSII_, which indicates the actual photochemistry efficiency under natural light conditions, was significantly (9.7%) higher (*P* < 0.05) in plants treated with NaCl than in the control plants at the beginning of the reproductive growth stage (Fig. 5b).

### Sequencing output and assembly

To investigate the molecular mechanisms that improve flower and seed development in *S. salsa* plants grown in the presence of NaCl, we collected flowers from control and NaCl-treated (NaCl) plants and subjected them to RNA-seq. After excluding low-quality sequences, we used the clean reads to generate a de novo transcriptome assembly using Trinity [46] and analyzed the differences in gene expression between control and NaCl-treated (NaCl) flowers after clustering by Corset [47]. The transcripts and genes used in this analysis were described by Guo et al. [48].

### Functional annotation of DEGs in control vs. NaCl-treated flowers

We analyzed differentially expressed genes (DEGs) in the flowers of control vs. NaCl-treated plants using the RSEM software package [49]. We performed functional annotation of DEGs between the two groups by BLAST analysis based on seven databases as described previously. To explore the biological functions of the DEGs, we mapped the DEGs to KEGG pathways. In total, 3640 DEGs were mapped to 124 KEGG pathways, such as “photosynthesis”, “starch and sucrose metabolism”, “fatty acid biosynthesis and elongation”, and “amino sugar and nucleotide sugar metabolism” [48].

### DEGs related to antenna proteins and photosynthesis in *S. salsa* flowers

**Fig. 6:**
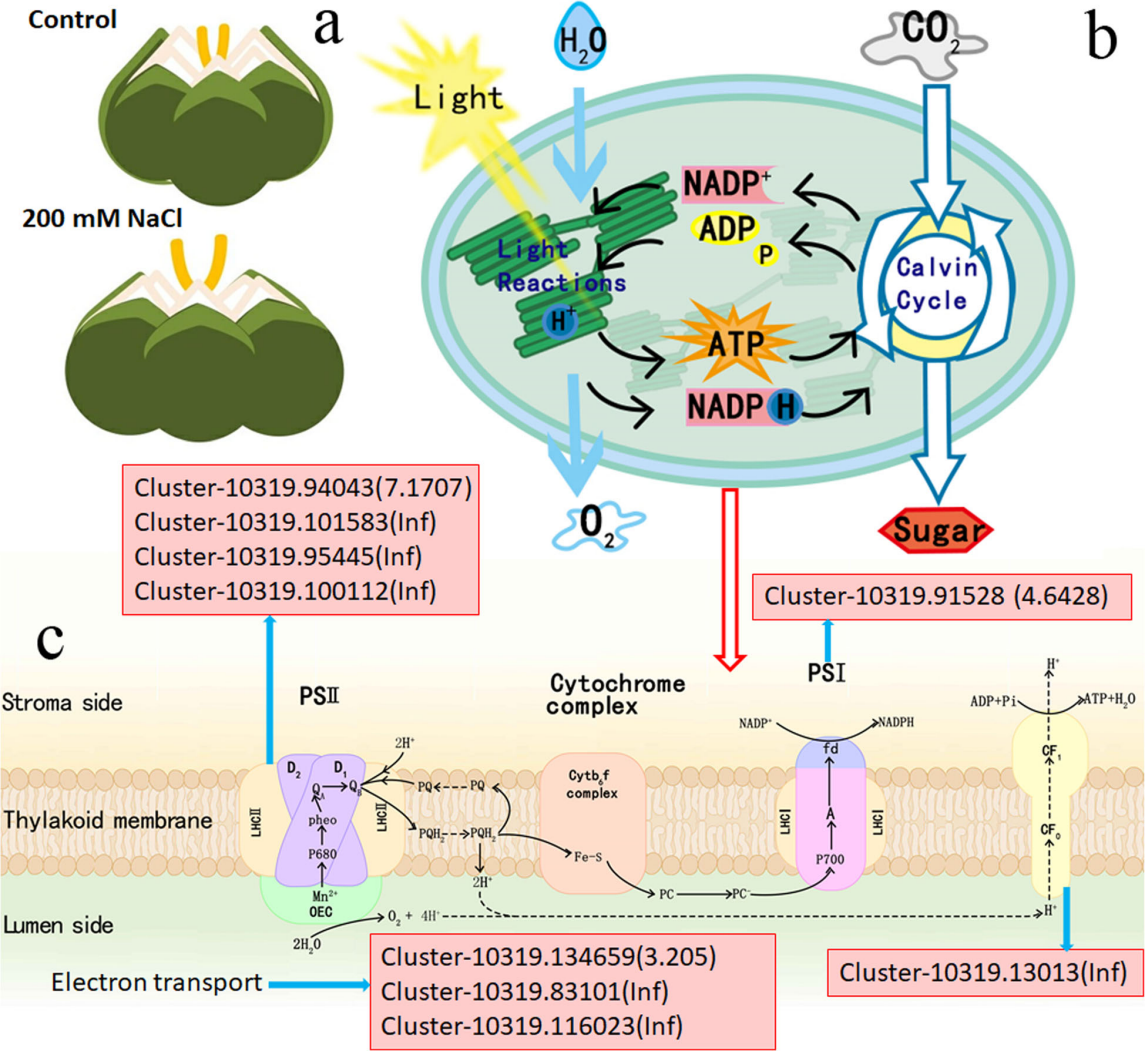
DEGs related to antenna proteins and photosynthesis in *S. salsa* flowers based on KEGG pathway analysis. Red indicates significantly increased expression of the corresponding DEGs in flowers from NaCl-treated vs. control plants based on the current RNA-seq data. (**a**) Flowers from control and NaCl-treated plants. (**b**) Chloroplasts from *S. salsa* plants. (**c**) Light energy capture and electron transfer. The value and Inf in parentheses indicate the log_2_ fold change and infinite in NaCl-treated plants compared to the control, respectively

Light harvesting is the first step in photosynthesis. Light-harvesting complexes (LHCs) containing the photosynthetic pigments chlorophyll and carotenoid, the most important structures in this process, are located near PSI (photosystem I) and PSII [50, 51]. LHC proteins are encoded by a multi-gene family in the nucleus [52]. Plants contain 14 types of LHC proteins (Lhca1–Lhca6 and Lhcb1–Lhcb8). Lhca-type proteins are located near the PSI reaction center, whereas Lhcb-type proteins are located near the PSII reaction center [53]. In the present study, six DEGs were identified in *S. salsa* flowers that mapped to antenna proteins, of which four were upregulated and two were downregulated. Compared to the control, the four DEGs encoding Lhcb1 were upregulated in the flowers of NaCl-treated plants, including three that were highly expressed under NaCl treatment (Fig. 6). However, DEGs encoding Lhcb4 and Lhcb5 were downregulated in flowers in the NaCl-treated group (Fig. 6).

In addition to light harvesting, electron transfer is also a prerequisite for the completion of photosynthesis, especially under unfavorable environmental conditions. Salt stress significantly inhibits photosynthetic efficiency in plants [54, 55]. The photosynthetic electron transport chain in plants is composed of four protein complexes: PSII, the cytochrome (Cytb6f) complex, PSI, and ATP synthase. We identified 28 DEGs related to photosynthesis in the flowers of control vs. NaCl-treated plants, which may have led to functional changes in photosynthesis under high-salinity conditions (Additional file 5: Figure S5).

PSII consists of various types of chlorophyll binding components. This complex is responsible for light harvesting and electron transport during photosynthesis immediately after water oxidation [56]. PSI, a chlorophyll-protein complex, catalyzes the transfer of electrons from plastocyanin/cytochrome c6 to ferredoxin/flavodoxin, a process driven by light [57]. The PSI subunits PsaF and PsaN interact with ferredoxin or plastocyanin [58]. In the flowers of NaCl-treated *S. salsa* plants, DEGs encoding PsaF were downregulated. By contrast, of the two DEGs related to PsaN, one was upregulated and one was downregulated (Fig. 6). Moreover, five DEGs related to the gamma chain of ATP synthase were identified, including one that was upregulated and four that were downregulated (Fig. 6).

### DEGs related to carbon utilization and sucrose and starch metabolism in *S. salsa* flowers

In addition to DEGs related to light harvesting and electron transfer, we examined the expression of DEGs related to carbon utilization in flowers from the two groups of *S. salsa* plants. We identified 35 upregulated DEGs and 12 downregulated DEGs in the flowers of NaCl-treated plants compared to the control (Additional file 5: Figure S5). Of these, 16 genes were expressed at high levels specifically in flowers from NaCl-treated plants compared to the control. Detailed information is provided in Additional file 6: Table S1.

**Fig. 7:**
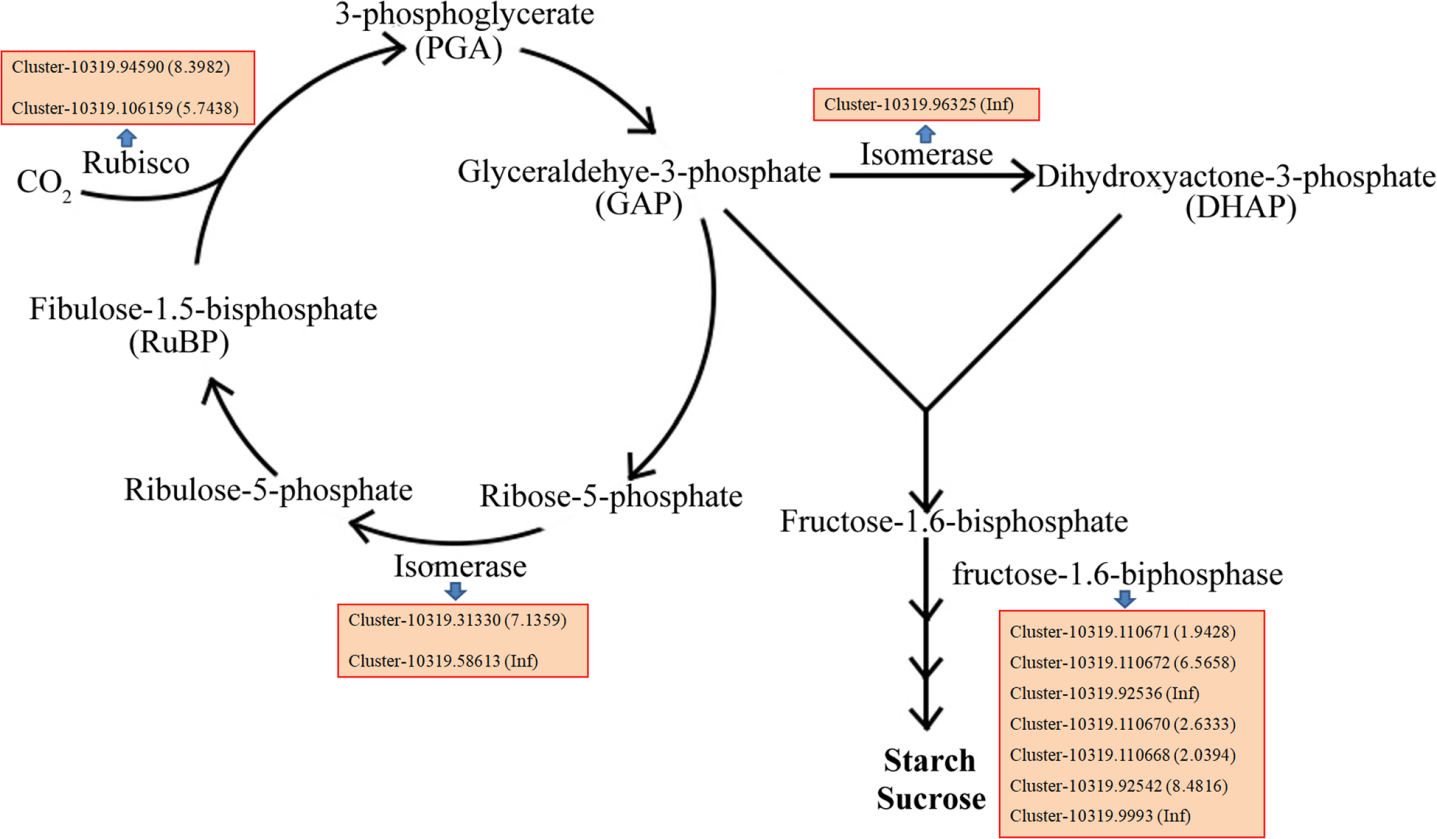
Upregulated DEGs involved in CO_2_ assimilation in the flowers of NaCl-treated vs. control plants based on the current RNA-seq data. The value and Inf in parentheses indicate the log_2_ fold change and infinite in NaCl-treated plants compared to the control, respectively

Carboxylation is the first step in the CO_2_ assimilation process. Ribulose-1,5-bis-phosphate carboxylase/oxygenase (Rubisco, EC 4.1.1.39) is the key enzyme in this process, which links organic and inorganic matter in the biosphere [59]. Two DEGs (Cluster-10,319.94590 and Cluster-10,319.106159) encoding Rubisco were upregulated in the flowers of NaCl-treated plants (Fig. 7). The increased CO_2_ fixation efficiency in *S. salsa* plants treated with NaCl might be due to the enhanced expression of genes encoding Rubisco. Similarly, in *Dunaliella salina*, photosynthesis-related genes are upregulated at the optimum NaCl concentration (1.7 M), which is associated with optimum growth [60]. During the regeneration process of ribulose 1,5-diphosphate (RuBP), two DEGs (Cluster-10,319.31330 and Cluster-10,319.58613) encoding isomerases were upregulated in the flowers of NaCl-treated plants. Seven DEGs (Cluster-10,319.110671, Cluster-10,319.110672, Cluster-10,319.92536, Cluster-10,319. 110,670, Cluster-10,319.110668, Cluster-10,319.92542, and Cluster-10,319.9993) were upregulated in flowers from NaCl-treated plants (Fig. 7). These results indicate that the higher sugar contents in flowers and the higher starch contents in ovules are accompanied by the upregulated expression of the associated genes and high photosynthetic efficiency (Fig. 3a).

**Fig. 8:**
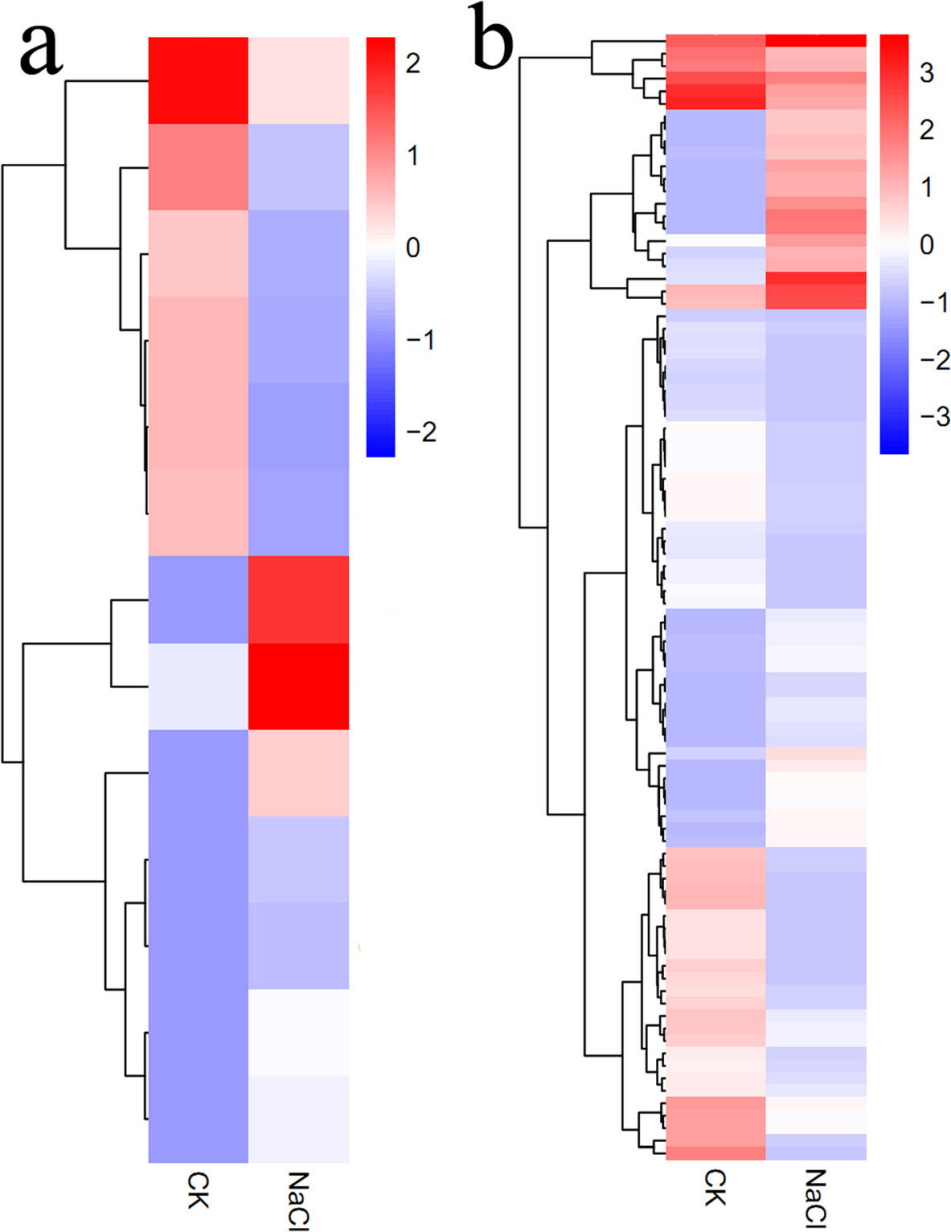
Heat map cluster analysis of DEGs annotated to sucrose metabolism (**a**) and starch metabolism (**b**) pathways in the flowers of control and NaCl-treated plants. Red represents upregulated genes and blue represents downregulated genes, with log_2_ (fold change) = 2.0

Starch is an essential source of carbon for plant development, especially in floral organs during reproductive growth. To investigate the reason for the different starch and sugar contents in *S. salsa* ovules and flowers in the two treatment groups, we examined the expression of DEGs involved in sugar and starch metabolism. We identified 133 DEGs related to sugar metabolism and sugar transport in *S. salsa* flowers (Fig. 8a). These DEGs might participate in sugar binding, sugar transport, and sugar phosphorylation, including 77 upregulated and 56 downregulated DEGs. We also identified 94 DEGs involved in starch metabolism in *S. salsa* flowers (Fig. 8b; detailed information is provided in Additional file 8: Table S2). These genes, which may participate in starch binding, starch metabolic processes, and starch phosphorylation, included 55 upregulated and 39 downregulated DEGs. Finally, 30 and 27 DEGs involved in sugar and starch metabolism, respectively, were expressed at significantly higher levels (*P* < 0.05) in flowers from NaCl-treated vs. control plants; this upregulation likely increased the sugar and starch contents in these plants.

Sucrose synthetase (SS, EC: 2.4.1.13), sucrose phosphate synthase (SPS, EC: 2.4.1.14), fructokinase (EC: 2.7.1.4), and phosphoglucomutase (PGM1, EC: 5.4.2.2) are key enzymes in sucrose and starch metabolism. SS plays a vital role in the conversion of glucose and fructose to sucrose, as do SPS and PGM1. Based on our RNA-seq data, genes involved in this pathway were expressed at higher levels in *S. salsa* flowers from NaCl-treated vs. control plants, such as genes encoding SPS (two upregulated DEGs), SS (eight upregulated and two downregulated DEGs), and PGM1 (two upregulated DEGs). These findings are consistent with the high sugar content in the flowers of NaCl-treated vs. control plants (Additional file 7: Figure S6).

### Verification of RNA-seq data using qRT-PCR

**Fig. 9:**
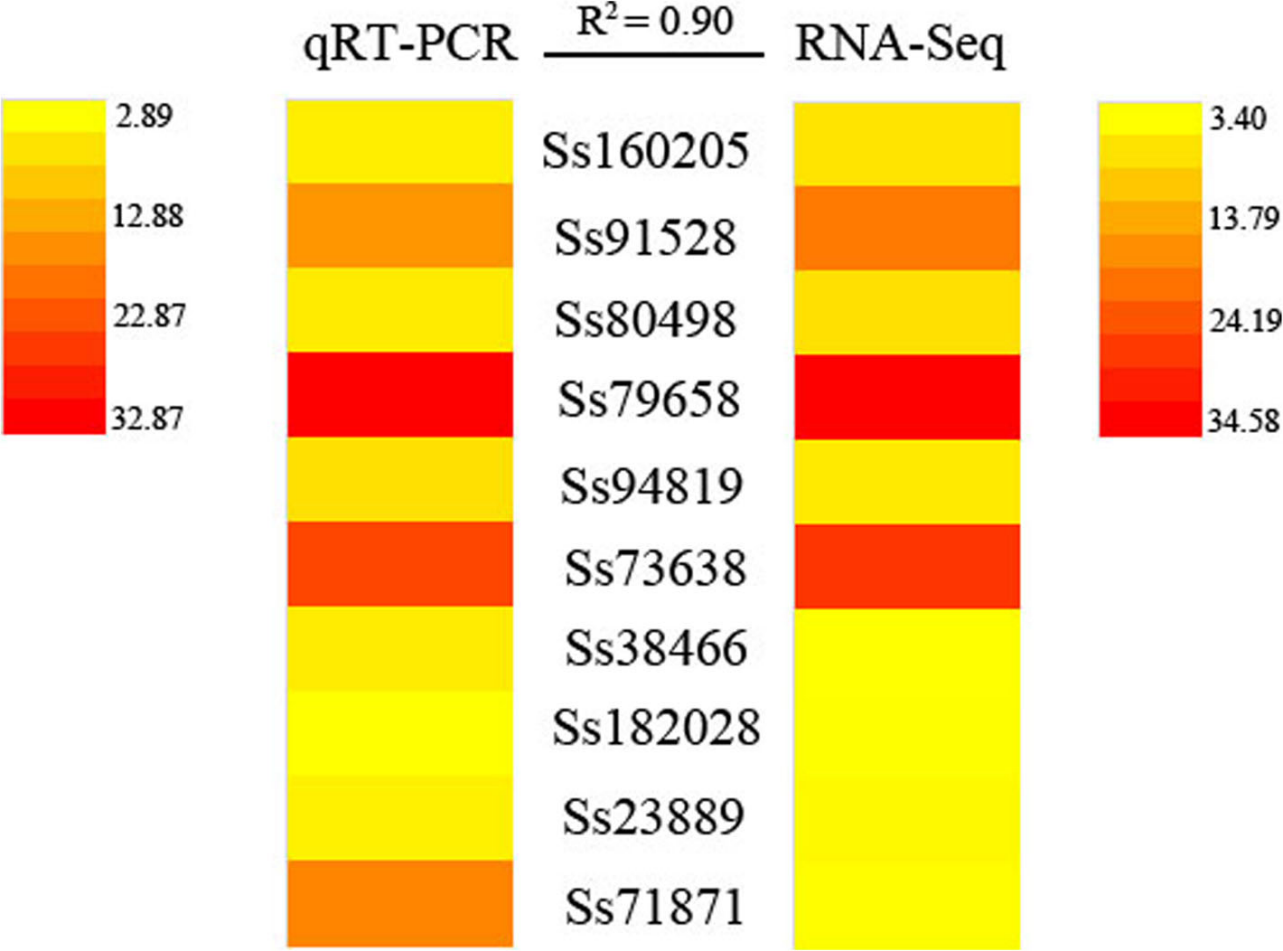
Validation of RNA-seq results by qRT-PCR. The expression levels of ten randomly selected genes in *S. salsa* flowers from control and NaCl-treated plants were detected using qRT-PCR. *R*^2^ represents the correlation coefficient between the two platforms. Primers are listed in Additional file 9: Table S3

To confirm the changes in gene expression detected by RNA-seq, we subjected ten randomly selected DEGs to quantitative qRT-PCR. The results from qRT-PCR are in close agreement with the RNA-seq data, with a correlation coefficient of *R*^2^ = 0.90 (Fig. 9), thus verifying the reliability of the RNA-seq results.

## Discussion

A low concentration of salt in the growth medium can seriously inhibit the reproduction of non-halophytes, as well as the vegetative growth [21, 61–64]. This inhibitory effect is primarily due to the accumulation of Na^+^ and Cl^−^ in reproductive organs [61]. Salinity strongly inhibits the formation of reproductive organs in *Arabidopsis thaliana* [65], decreases the fertility of *Sorghum bicolor* [66], and reduces pollen tube growth in grapevine styles [23], thereby reducing seed number [67]. Reproduction drastically decreases in Arabidopsis plants treated with 200 mM NaCl for only 12 h, with a very low percentage of seed formation (5% of ovules) [68]. However, for the euhalophyte *S. salsa*, the addition of 200 mM NaCl to the growth medium promotes reproduction, as reflected by the significant increase in seed yield and seed size [28], an effect exactly opposite that in non-halophytes treated with NaCl [69].

Total soluble sugars help plants adjust the osmotic potential, increase water uptake under high-salinity conditions [70], and provide raw materials for plant growth. In *Chenopodium quinoa* Willd, NaCl treatment reduces the sugar content in the embryonic axis [71]. By contrast, in the present study, the total soluble sugar contents increased not only in leaves and stems, but also in flowers of *S. salsa* treated with 200 mM NaCl (Fig. 2). Abiotic stresses such as high NaCl inhibit starch accumulation and cause dramatic yield losses in crops [72] such as the rice [73]. However, in the halophyte *S. salsa*, starch levels in the embryo sac significantly increased (*P* < 0.05) in response to 200 mM NaCl treatment compared to those in control plants (Fig. 1a, b); the accumulated starch supplies nutrients for the developing embryo and endosperm [45, 74, 75]. The high starch levels represent a good source of material for ovule and seed development in NaCl-treated *S. salsa* plants, which may be attributed to high soluble sugar contents in flowers and leaves (Fig. 2). In turn, soluble sugar accumulation benefits from the high efficiency of photosynthesis in leaves (Fig. 3a) and in flowers (Additional file 4: Figure S4) in *S. salsa* plants treated with NaCl.

During the flowering period, reproductive structures become major sink organs and require more photosynthetic products than other organs. The photoassimilates will be provided mainly by the leaves because chlorophyll content, chloroplasts and photosynthesis rate in *S. salsa* flowers were all much lower than those in leaves, although 200 mM NaCl significantly enhanced the chlorophyll content and the photosynthetic efficiency both in flowers and leaves (Figure S2, S3, S4, Figs. 3 and 4) [76]. However, plant growth is inhibited, the chlorophyll levels and photosynthetic efficiency decrease in the non-halophyte such as mung bean and Chinese cabbage under NaCl treatment [77, 78]. In the present study, NaCl treatment significantly (*P* < 0.05) promoted the photosynthetic oxygen evolution capacity in petals and the photosynthetic efficiency in leaves of *S. salsa* plants (Additional file 4: Figure S4 and Fig. 3a), leading to a greater accumulation of photosynthetic products in NaCl-treated vs. control plants. These results perhaps explain why seeds from 200 mM NaCl-treated *S. salsa* were significantly larger (*P* < 0.05) than those of the control (Additional file 1: Figure S1).

To investigate the molecular mechanism by which NaCl improves seed development in *S. salsa*, we performed RNA-seq of the flowers of 0 and 200 mM NaCl-treated plants. Numerous DEGs were identified between control and NaCl-treated plants, including many involved in photosynthesis and carbohydrate metabolism (Additional file 8: Table S2). Light harvesting is the first step in photosynthesis, and LHCs are the most important structures in these processes [50, 51]. In the present study, four out of six light-harvesting-related genes were upregulated in NaCl-treated flowers (Fig. 6); the increased expression of these genes enhances the light-harvesting ability of the plant. Electron transfer is another prerequisite for the completion of photosynthesis, especially under unfavorable environmental conditions. Salt stress significantly inhibits the photosynthetic efficiency of crops such as rice [54, 55]. We identified 28 photosynthesis-related DEGs between NaCl-treated and control plants (Additional file 5: Figure S5), leading to differences in oxygen releasing efficiency in petals between the two groups of *S. salsa* (Additional file 4: Figure S4).

PSI and PSII are the main components of the photosynthetic apparatus in plants [56, 57], including the proximal antenna of PSII (cp43) [79] and the PSI subunits PsaF and PsaN [58]. In the present study, DEGs related to photosynthesis, such as genes encoding PsbP and PsbR, were downregulated in NaCl-treated *S. salsa* flowers (Fig. 6). In these plants, DEGs encoding PsaF were downregulated, whereas one DEG encoding PsaN was upregulated and the other was downregulated (Fig. 6). One DEG related to the gamma chain of ATP synthase was upregulated and the four other genes were downregulated (Fig. 6). Meanwhile, the *P*_n_ was higher in NaCl-treated than in control leaves (Fig. 3a). Perhaps the increased photosynthetic parameters were associated with the upregulated genes in plants treated with NaCl. Indeed, the increased growth of *Dunaliella salina* is associated with upregulated expression of photosynthesis-related genes at the optimum NaCl concentration (1.7 M) [60].

Carbon utilization is an important process in metabolism. Starch is an essential carbon source for plant growth and development, especially in floral organs during reproductive growth [80]. The high efficiency of carbon utilization and high levels of starch accumulation in the flowers of NaCl-treated plants may benefit from the high efficiency of photosynthesis and carbohydrate metabolism; these factors are inseparable from the high expression levels of genes related to photosynthesis [60]. Among the 47 DEGs in the carbon utilization pathway, 35 were upregulated and 12 were downregulated in the flowers of NaCl-treated plants compared to the control (Additional file 5: Figure S5). The enhanced efficiency of carbon utilization may be associated with the large number of highly expressed genes in the flowers of these plants. In non-halophytes, starch degrades into soluble sugars to help plants survive in unfriendly environments, such as drought or salt stress [81]. By contrast, in euhalophytes, the osmotic potential of the plant adjusts to high-salinity environments via the accumulation of large amounts of inorganic salt ions [82]. Thus, starch degradation may decrease in euhalophytes grown in the presence of NaCl. Together with enhanced carbon utilization, starch levels were higher in *S. salsa* vs. control plants. Similarly, in sweet sorghum, the high sugar content in leaves is associated with specific gene expression patterns under NaCl treatment [83].

Starch is a major material obtained from photosynthesis that is synthetized from sucrose in leaves [84]. The starch and sugar contents were significantly higher in *S. salsa* ovules and flowers than in control plants (Figs. 1 and 2). At the same time, genes involved in sugar and starch metabolism were differentially expressed. We identified 77 upregulated and 56 downregulated genes that might participate in sugar binding, sugar transport, and sugar phosphorylation in the flowers of NaCl-treated plants (Fig. 8a). We also identified 55 upregulated and 39 downregulated genes in the NaCl-treatment group that might participate in starch metabolic processes (Fig. 8b). For example, genes encoding sucrose synthetase, sucrose phosphate synthase, fructokinase, and phosphoglucomutase were expressed at high levels in the flowers of NaCl-treated plants (Additional file 5: Figure S5). These upregulated genes might be beneficial for starch accumulation in *S. salsa* and help reduce the decomposition of starch, thereby increasing the starch content. In summary, the altered expression levels of starch-related genes in the flowers of *S. salsa* plants treated with NaCl might lead to increased sugar and starch contents in flowers and ovules due to increased starch accumulation and reduced starch decomposition, thereby resulting in increased seed size.

The leaf is the main photosynthetic organ in green plants. Most photoassimilates are exported from the leaf to other growing organs, except for the basic levels needed to maintain daily metabolism. Seed filling ability is affected by the translocation of carbohydrates from photosynthetic organs (source, such as leaves) to storage organs (sink, such as seeds). In plants, polysaccharides are the dominant storage carbohydrates, which primarily exist in the form of starch and sucrose [85]. Sucrose is the preferred transported form of carbohydrates in plants, as it can be transported over long distances. During long-distance transport, sucrose is actively loaded from mesophyll cells into companion cell. The sucrose is then translocated to storage organs, such as roots and developing seeds. The photoassimilates in the phloem are exported to the receiving cells of the sink organ. The most common storage form of carbohydrates in developing seeds is starch; sucrose is generally converted to starch in developing organs.

In addition to leaves, floral organs with green tissues (such as green petals during early floral development) can also perform photosynthesis (Additional file 4: Figure S4) and produce photoassimilates (although which is much lower than that of leaves), and the photoassimilates could be translocated to nearby regions of the developing flower and seeds. This is consistent with the chlorophyll content in its floral organs (Additional file 2: Figure S2). The differential expression of genes involved in sucrose synthesis and allocation is consistent with the high sugar content in the flowers (Additional file 7: Figure S6) and the high starch content in the ovules of NaCl-treated plants (Fig. 1).

## Conclusion

**Fig. 10:**
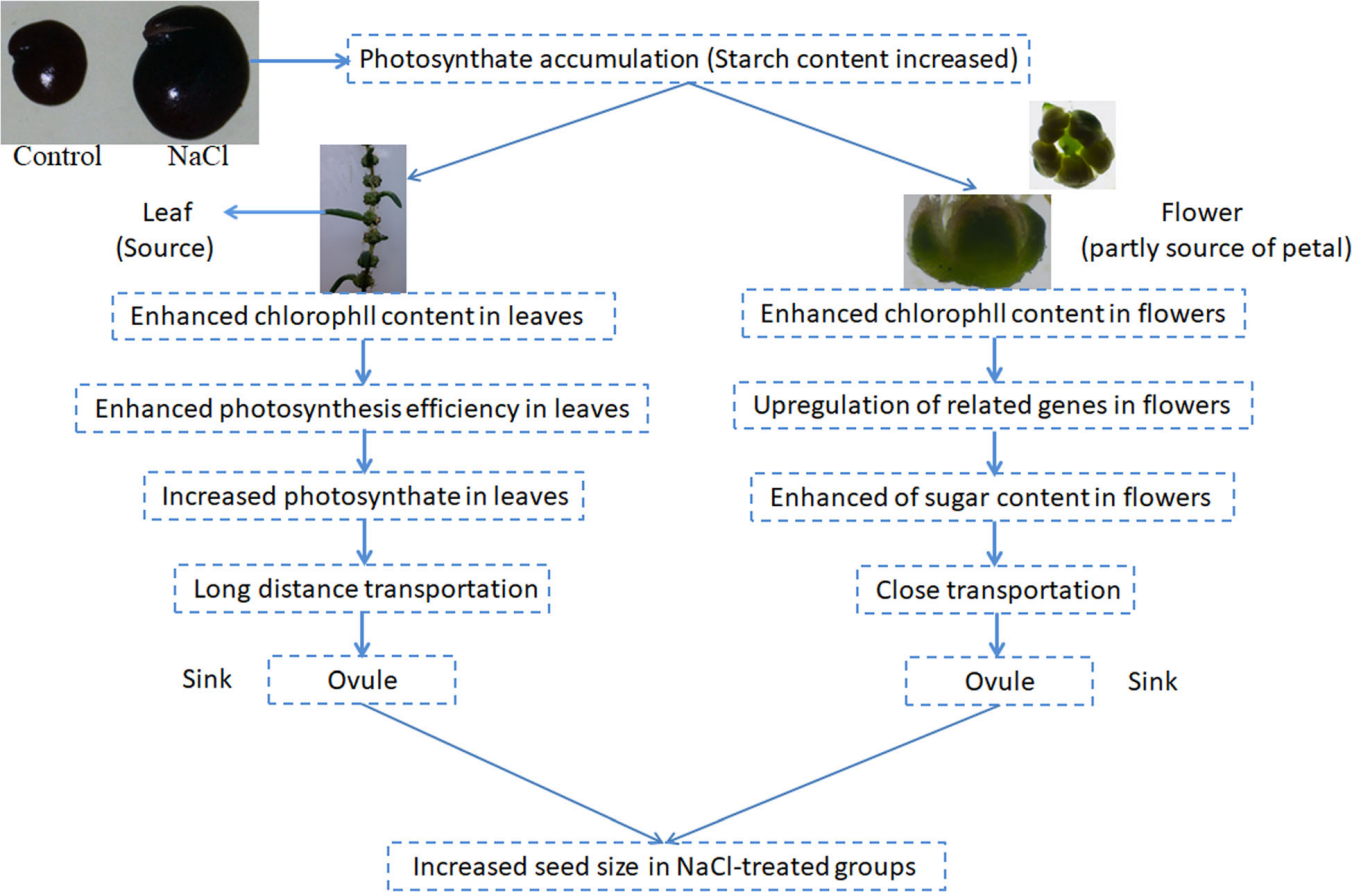
Working model of seed development in *S. salsa* under high-NaCl conditions. The enhanced seed size in *S. salsa* exposed to NaCl is mainly dependent on the supply of materials from leaves and petals, which provide increased levels of photosynthates for seed development, including increased sugar contents in flowers and starch contents in ovules. The enhanced sugar and starch accumulation in flowers and seeds is related to the upregulated expression of genes involved in photosynthesis, carbon utilization, and sucrose and starch metabolism, thus increasing seed size in NaCl-treated *S. salsa* plants

*S. salsa* seeds from plants grown in the presence of NaCl were significantly larger and more numerous than seeds from control plants. To investigate the mechanism underlying this difference, we measured the sugar and starch contents in the flowers and ovules of this euhalophyte for the first time, revealing increased levels of both products in *S. salsa* plants treated with NaCl. The improved reproductive growth, especially seed growth and development, in the euhalophyte *S. salsa* under salinity treatment requires more raw materials in the form of photosynthates. Two sources of photosynthates are provided for seed development in *S. salsa*. The major photosynthate provider is photosynthesis in leaves; these photosynthates are transported over a long distance to seeds. In addition, a small amount of photosynthesis occurs in the petals of *S. salsa* flowers; these photosynthates are transported to seeds over a short distance. The net photosynthesis rate was significantly higher in the leaves of *S. salsa* plants treated with NaCl than in untreated control plants, and this was accompanied by increased chlorophyll contents in the leaves and flowers of plants treated with NaCl. The higher efficiency of photosynthesis and higher levels of photosynthates in *S. salsa* plants treated with NaCl are associated with the expression levels of genes involved in light harvesting, electronic transport, and photosynthesis and genes encoding the enzymes in the sugar and starch metabolism pathway. We propose a model for the possible mechanism leading to increased seed size in *S. salsa* plants treated with NaCl (Fig. 10). The RNA-seq dataset produced in the present study represents an important resource for further investigating the enhanced sugar and starch contents in the flowers of *S. salsa* plants in the presence of NaCl. Further investigations of the underlying molecular mechanism should shed light on the precise reason for the high carbohydrate content in *S. salsa* flowers under salt treatment.

## Methods

### Samples

*Suaeda salsa* L. seeds were obtained from Dongying Research Academy of Agriculture Science with the permissions of Dongying government, and were stored in a refrigerator (< 4 °C) before use as described in Guo et al. [86]. *S. salsa* is a wild plant resource that widely distributed in natural saline habitats in the north of China. The collection of seeds and the performance of experimental research on such plant were complied with the national guidelines of China.

Seeds were sown in pots filled with sand and subjected to non-saline control treatment (Hoagland nutrient solution without NaCl) and 200 mM NaCl treatment (200 mM NaCl dissolved in Hoagland nutrient solution). Twelve pots of plants were subjected to each treatment, with two plants maintained per pot. The NaCl treatments were performed from the sowing stage until mature seed production using the growth conditions described in Guo et al. [28]. Salt was added progressively, so as not to produce any osmotic stress to the growth of the plant [87]. During the growth period, a relatively constant NaCl concentration in the sand was maintained by applying solutions twice per day using three times the volume of the water-holding capacity of the pot. All experiments described in the paper were performed at Shandong Normal University with the permission of Dongying Research Academy of Agriculture Science (China).

### Analysis of seed development

In natural habitats, *S. salsa* produces two types of seeds in the same plant, black seeds and brown seeds; the former develop and mature earlier than the latter. During the reproduction period, both types of seeds were harvested from the same position on each branchlet (e.g., the first branches that formed on the main stems, branch 1 vs. branch 1, counted from the plant bottom) of plants in the two treatment groups (control and 200 mM NaCl treatment), and fruit size was measured with a micrometer.

### Analysis of ovule development

To measure starch accumulation in ovules, Lugol’s staining was performed as described by Mizzotti et al. [45]. Pistils were isolated from the flowers before and after fertilization and immediately fixed in FAA solution (50% ethanol: acetic acid: formaldehyde, 18: 1: 1, by volume) for at least 24 h. The pistils were cleared with 1.0 M NaOH for 12 h, washed at least three times with distilled water, and stained with Lugol’s solution. Stained pistils were observed under a Nikon 80i Eclipse × 200 DIC microscope.

### Measurement of TSS contents

To quantify TSS contents in control and 200 mM NaCl-treated plants, the leaves, the corresponding stem, and flowers from the same branches were harvested at 105 DAS and were dried. 50 mg oven-dried samples were ground to a powder with liquid nitrogen. TSS was extracted in 80% ethanol and quantified using the classical anthrone method [88] in a UV-visible spectrophotometer (UV-1700, Shimadzu, Japan). A standard curve was constructed using glucose, and the results were expressed as the percentage of TSS vs. dry weight.

### Determination of photosynthesis parameters

At the beginning of the reproductive growth phase (103 DAS), expanded leaves of control and 200 mM NaCl-treated plants were selected and used to measure net photosynthetic rate (*P*_n_), stomatal conductance (*g*_s_), transpiration rate (*E*), and intercellular CO_2_ concentration (*C*_i_) using a portable photosynthetic tester (CIRAS-3, PP, USA). The chlorophyll fluorescence parameters were determined using a portable fluorometer (FMS-2, Hansatech, UK) as described in Kooten and Snel [89]. To determine the minimum and maximum fluorescence, mature leaves were dark-adapted for 1 h before testing. Minimum fluorescence yield (*F*_0_, dark-adapted leaves) and maximum fluorescence yield (*F*_m_, dark-adapted leaves after a saturating pulse of 8000 μmol m^− 2^ s^− 1^ for 0.8 s) were recorded. Steady-state fluorescence (*F*_s_) and light-adapted-state fluorescence (*F*_m_′) parameters were determined after the leaves were illuminated with 500 photons μmol m^− 2^ s^− 1^ of activating light. The maximum photochemical efficiency of PSII was calculated using the formula: *F*_v_/*F*_m_ = (*F*_m_ – *F*_0_)/*F*_m_. The quantum yield of PSII was calculated using the formula: Φ_PSII_ = (*F*_m_′ – *F*_s_)/*F*_m_′ [90]. Fifteen repetitions from six individual control and NaCl-treated plants were performed.

The flowers at the early stage were collected from *S. salsa* plants grown with control and NaCl treatment, respectively, the petals were isolated and were used for determination of the oxygen evolution using the Clark-type Oxygen Electrode (Hansatech, Chlorolab2, UK).

### Measurement of chlorophyll contents in *S. salsa* leaves and flowers

At the beginning of the reproductive growth phase (103 DAS), expanded leaves and flowers (0.3 g fresh weight) of plants treated with 0 and 200 mM NaCl were collected, washed with distilled water, and incubated in 10 ml extraction solution (80% acetone: dimethyl sulfoxide, 1:1, by volume) in the dark for 24 h. The final volume was adjusted to 25 ml using 80% acetone. The light absorption values at 645 nm and 663 nm were determined with a spectrophotometer (DU2600, Beckman, Germany) as described by Sui et al. [83]. The chlorophyll content in the flowers was determined during early development (108 DAS) as described above. Chloroplasts in *S. salsa* petals were observed under a differential interference contrast microscope (DIC, ECLIPSE 80i, Nikon, Japan).

### Total RNA extraction, library construction, and sequencing

Before meiosis (108 DAS), flowers were collected from control and NaCl-treated plants. Total RNA extraction and detection were performed as described by Guo et al. [48]. Sequencing libraries were generated and processed as described by Hu et al. [91]. After cluster generation, the libraries were sequenced on the Illumina HiSeq 4000 platform (Novogene Bioinformatics Technology Co. Ltd., Beijing, China). Three biological replicates were used for RNA-seq.

### Transcriptome assembly and functional annotation of genes

Raw reads were cleaned by removing low-quality sequences, sequences containing vector adapter, and poly-N. De novo transcriptome assembly was performed using Trinity [46], and cluster analysis was performed using Corset [47]. The assembled unigenes expressed in *S. salsa* flowers were annotated as described by Guo et al. [48].

### Analysis of DEGs

DEGs in *S. salsa* flowers from NaCl-treated plants vs. control were analyzed using the DESeq R package (1.10.1). The processing and screening criteria were as described by Guo et al. [48].

### Quantitative real-time PCR

To verify the DEGs identified by RNA-seq, ten DEGs were randomly selected and subjected to qRT-PCR (quantitative real-time PCR) using specific primers designed with Beacon Designer software (version 7.0); the primers are listed in Additional file 9: Table S3. The procedures and calculations were performed as described in Guo et al. [48].

### Statistical analysis

Each experiment was performed on six randomly chosen replicate plants; the statistical results are presented as means ± standard deviation (SD). The values were analyzed by one-way ANOVA using SPSS (version 17). Different letters in the figures indicate a significant difference among means at a level of 0.05 based on Duncan’s test.

## Supplementary information

**Additional file 1: Figure S1.** Photograph (a) and length (b) of fresh black and brown seeds of *S. salsa* plants grown in sand in medium containing 0 and 200 mM NaCl.

**Additional file 2: Figure S2.** Chlorophyll contents in the flowers of *S. salsa* plants grown in sand in medium containing 0 and 200 mM NaCl during the early reproductive growth stage (108 DAS). Values are presented as the means ± SD of five replicates. Different letters in one group indicate a significant difference at *P* < 0.05 according to Duncan’s test.

**Additional file 3: Figure S3**. Observation of chloroplasts in the flower petals of *S. salsa* plants grown in sand in medium containing 0 (a) and 200 mM NaCl (b) during the early reproductive growth stage (108 DAS).

**Additional file 4: Figure S4.** Photosynthetic oxygen evolution in the petals of *S. salsa* plants grown in sand in medium containing 0 and 200 mM NaCl during the early reproductive growth stage (108 DAS). Values are presented as the means ± SD of three replicates. Different letters indicate a significant difference at *P* < 0.05 according to Duncan’s test.

**Additional file 5: Figure S5.** Number of DEGs annotated to photosynthesis and carbon utilization pathways in the flowers of control (0) and NaCl-treated (200 mM NaCl) *S. salsa* plants.

**Additional file 6: Table S1.** DEGs annotated to photosynthesis and carbon utilization pathways in the flowers of control (0) and NaCl-treated (200 mM NaCl) *S. salsa* plants.

**Additional file 7: Figure S6.** Unigenes predicted to be involved in sucrose and starch metabolism. DEGs were compared in flowers from NaCl-treated vs. control plants. Red boxes indicate significantly increased expression in NaCl-treated flowers compared to control flowers; yellow boxes indicate that some corresponding DEGs were upregulated and some were downregulated; black boxes indicate that the expression levels of the corresponding genes did not change.

**Additional file 8: Table S2.** DEGs annotated to sucrose and starch metabolism pathways in the flowers of control (0) and NaCl-treated (200 mM NaCl) plants.

**Additional file 9: Table S3.** Primer pairs used for quantitative RT-PCR.

## Data Availability

The data and materials that were analyzed in the current study could be available from the corresponding author on reasonable request.

## Abbreviations

PSII: Photosystem II
RNA-seq: RNA-sequencing
TSS: Total soluble sugars
DAS: Days after sowing
Pn: Photosynthetic rate
gs: Stomatal conductance
E: Transpiration rate
Ci: Intercellular CO_2_ concentration
F0: Minimum fluorescence yield
Fm: Maximal fluorescence yield
Fs: Steady-state fluorescence
Fm′: Light-adapted state fluorescence
DIC: Differential interference contrast microscope
ΦPSII: Actual photochemistry efficiency of PSII
DEGs: Differentially expressed genes
Fv/Fm: The maximum quantum yield of PSII
LHCs: Light-harvesting complexes
PSI: Photosystem I
Cytb6f: Cytochrome
Rubisco: Ribulose-1,5-bis-phosphate carboxylase/oxygenase
RuBP: Ribulose 1,5-diphosphate
SS: Sucrose synthetase
SPS: Sucrose phosphate synthase
PGM1: Phosphoglucomutase
