# Supplementary figures and images for: NaCl improves reproduction by enhancing starch accumulation in the ovules of the euhalophyte Suaeda salsa
Source: BMC Plant Biol. 2020 Jun 8;20:262. doi: 10.1186/s12870-020-02468-3 (PMC7282069; doi:10.1186/s12870-020-02468-3)

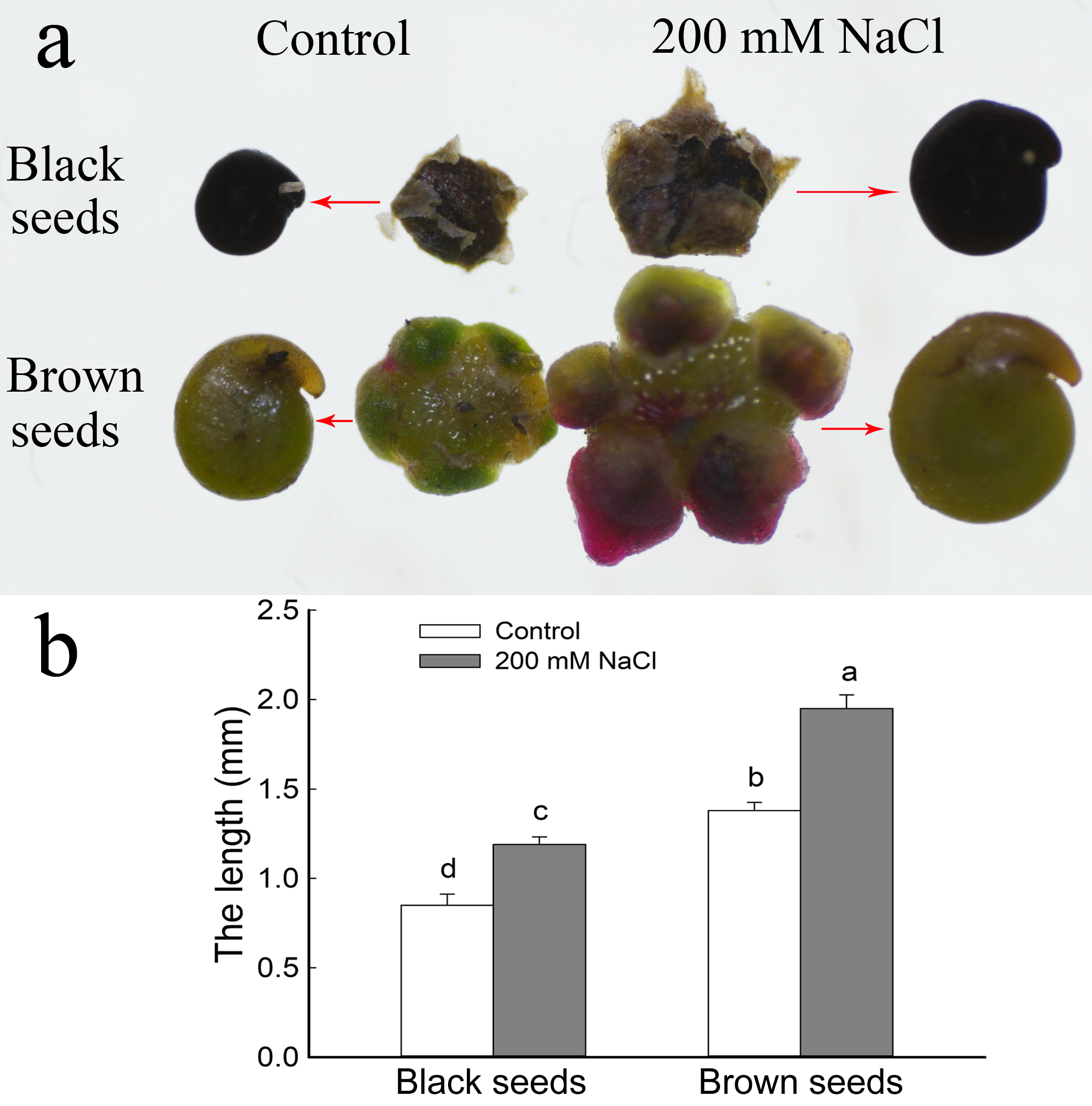

Supplement: Supplementary file 1 — Additional file 1: Figure S1. Photograph (a) and length (b) of fresh black and brown seeds of S. salsa plants grown in sand in medium containing 0 and 200 mM NaCl. [file 12870_2020_2468_MOESM1_ESM.tif]

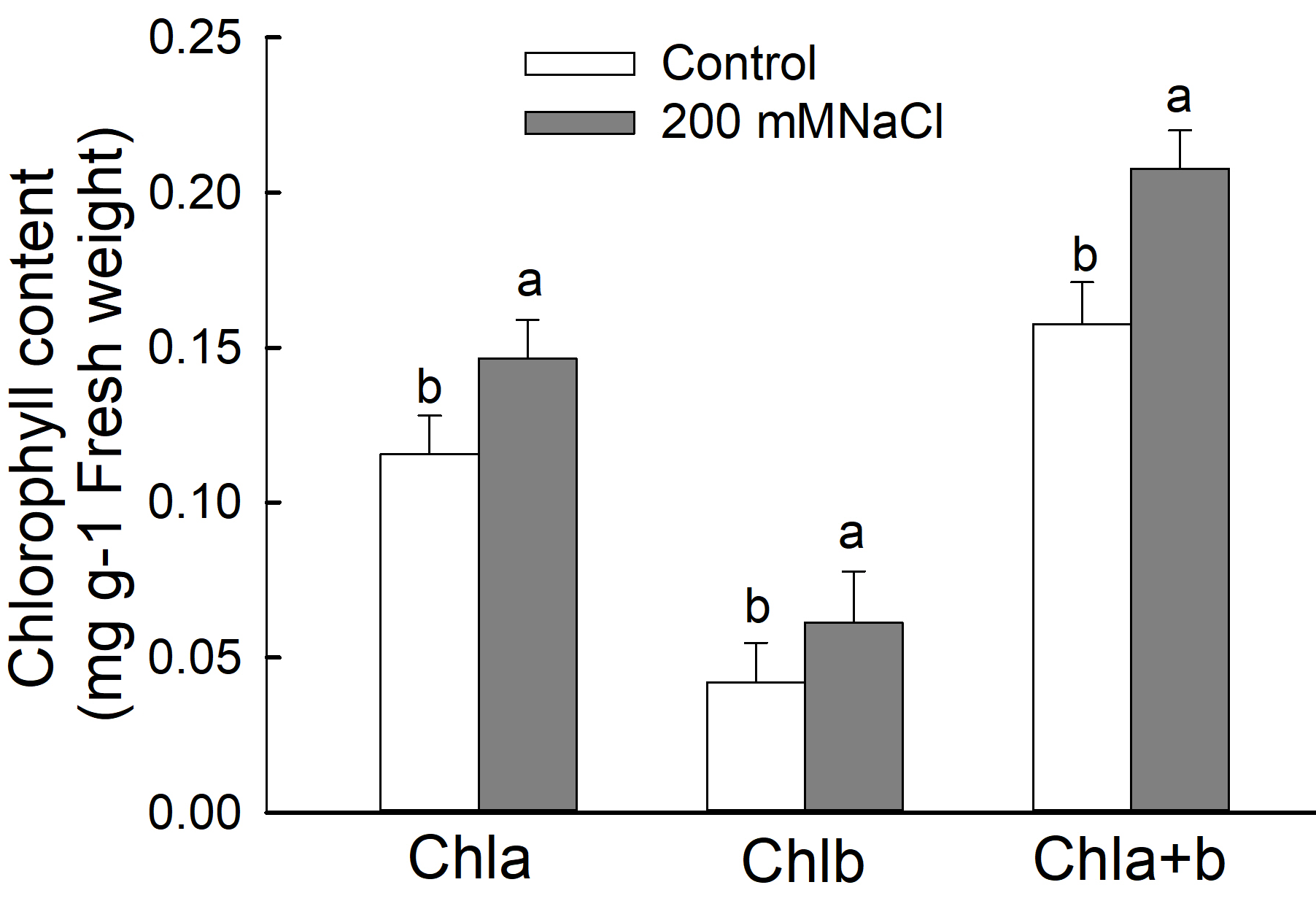

Supplement: Supplementary file 2 — Additional file 2: Figure S2. Chlorophyll contents in the flowers of S. salsa plants grown in sand in medium containing 0 and 200 mM NaCl during the early reproductive growth stage (108 DAS). Values are presented as the means ± SD of five replicates. Different letters in one group indicate a significant difference at P < 0.05 according to Duncan’s test. [file 12870_2020_2468_MOESM2_ESM.tif]

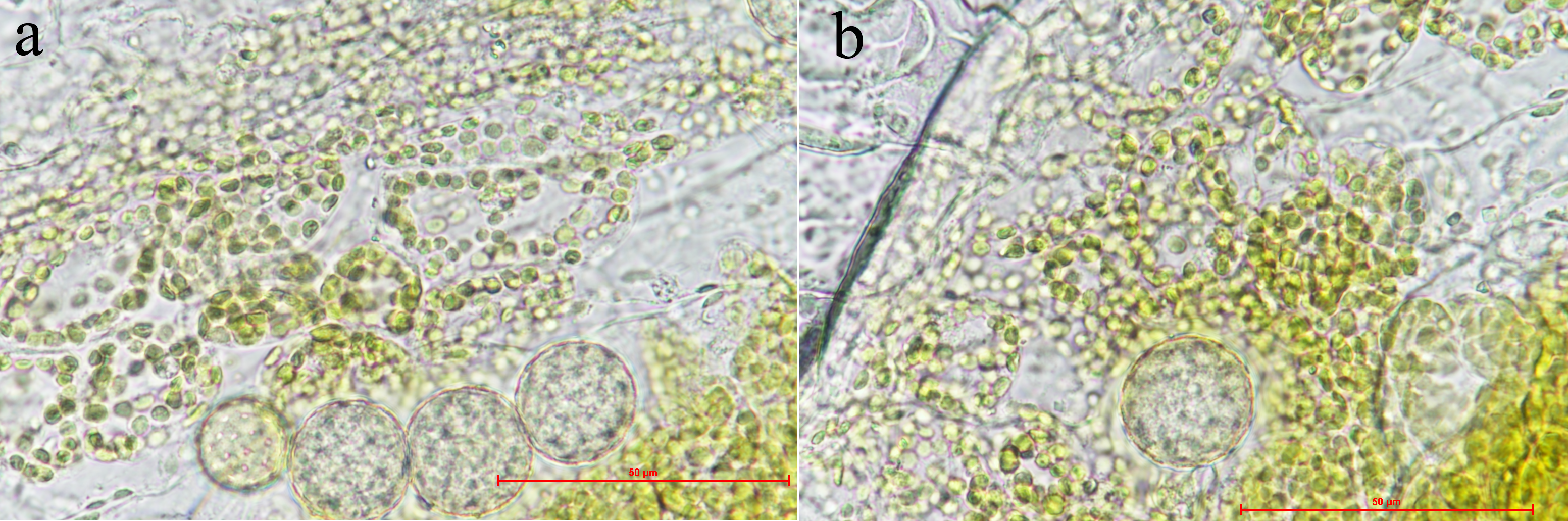

Supplement: Supplementary file 3 — Additional file 3: Figure S3. Observation of chloroplasts in the flower petals of S. salsa plants grown in sand in medium containing 0 (a) and 200 mM NaCl (b) during the early reproductive growth stage (108 DAS). [file 12870_2020_2468_MOESM3_ESM.tif]

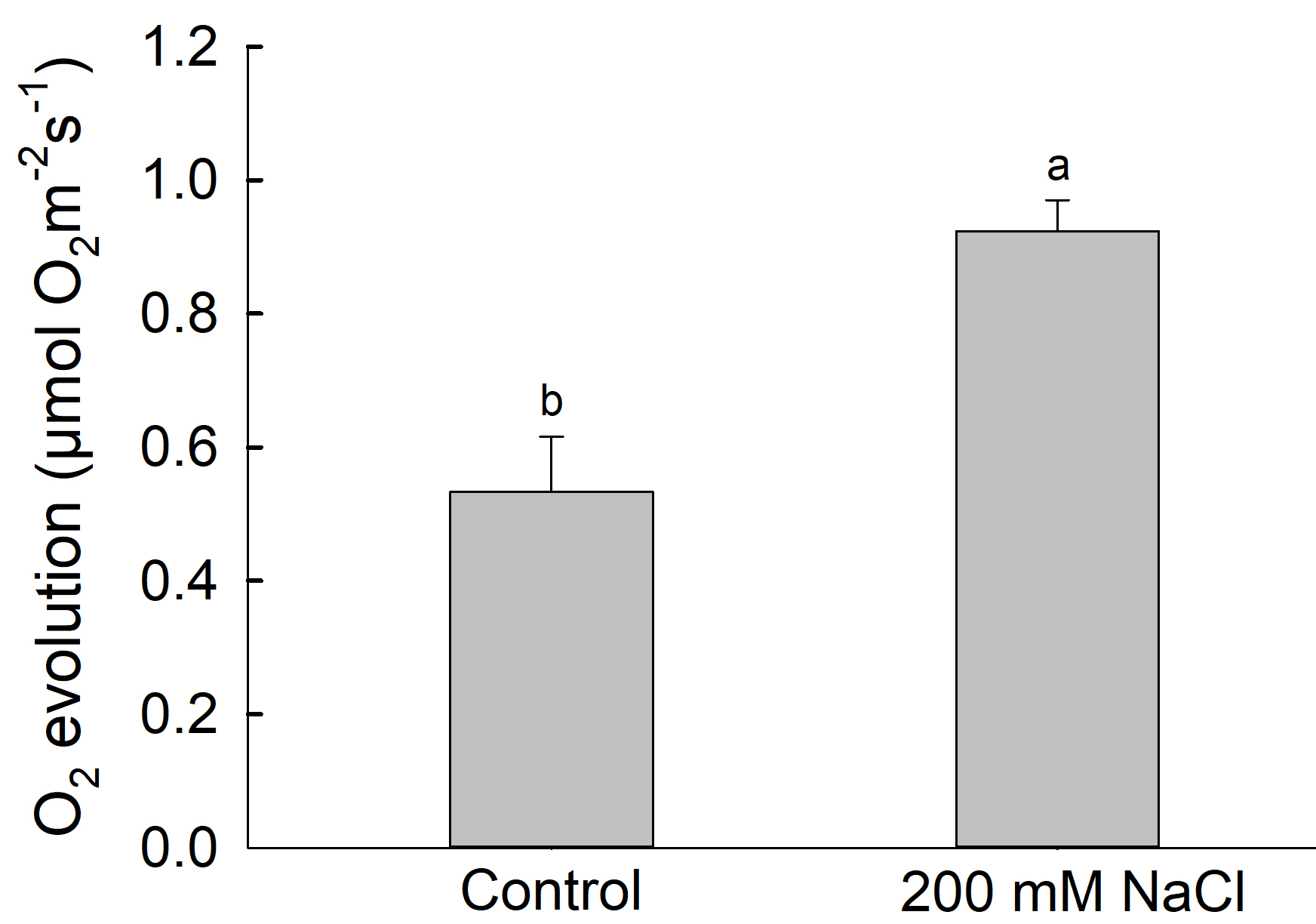

Supplement: Supplementary file 4 — Additional file 4: Figure S4. Photosynthetic oxygen evolution in the petals of S. salsa plants grown in sand in medium containing 0 and 200 mM NaCl during the early reproductive growth stage (108 DAS). Values are presented as the means ± SD of three replicates. Different letters indicate a significant difference at P < 0.05 according to Duncan’s test. [file 12870_2020_2468_MOESM4_ESM.tif]

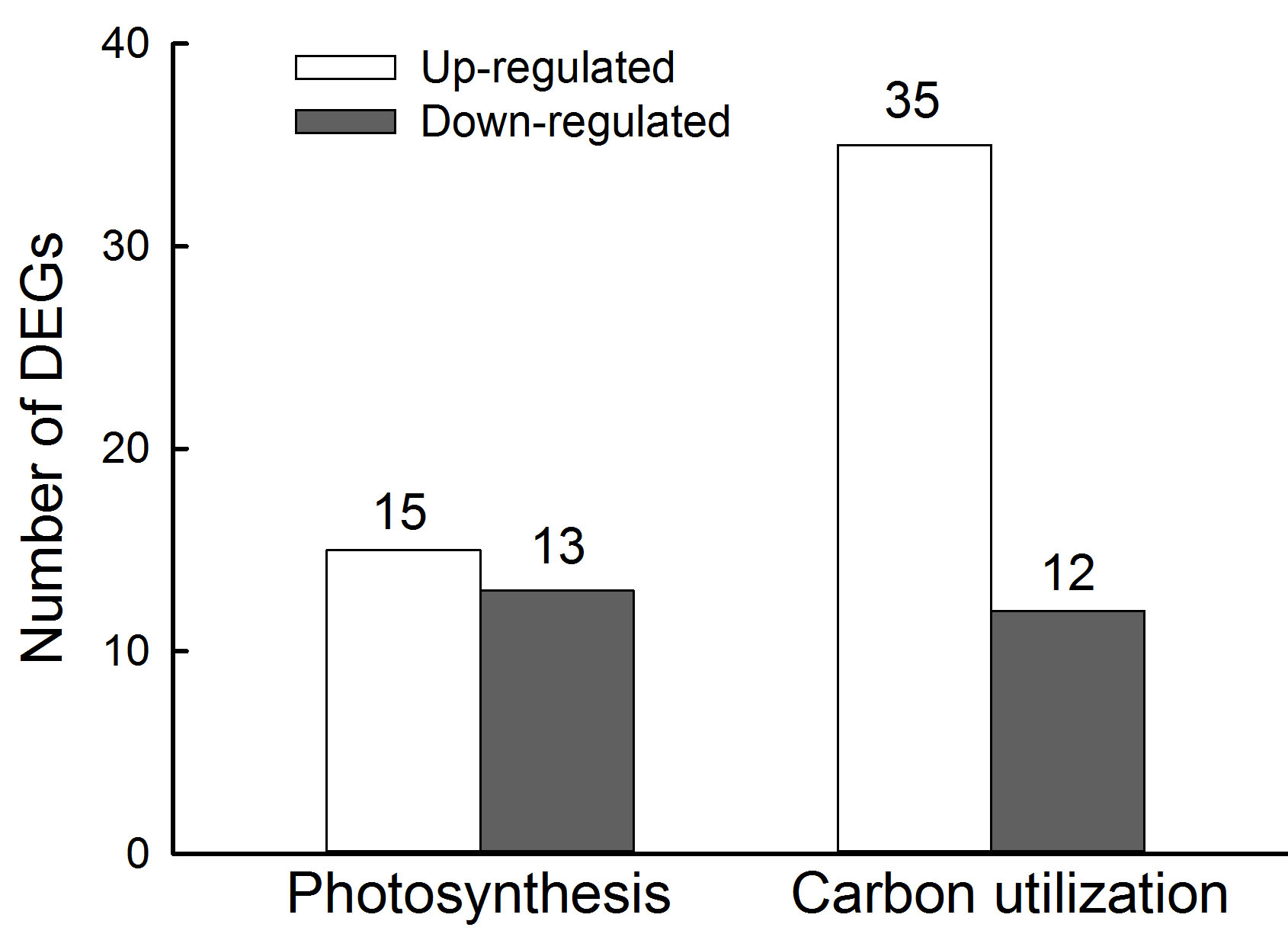

Supplement: Supplementary file 5 — Additional file 5: Figure S5. Number of DEGs annotated to photosynthesis and carbon utilization pathways in the flowers of control (0) and NaCl-treated (200 mM NaCl) S. salsa plants. [file 12870_2020_2468_MOESM5_ESM.tif]

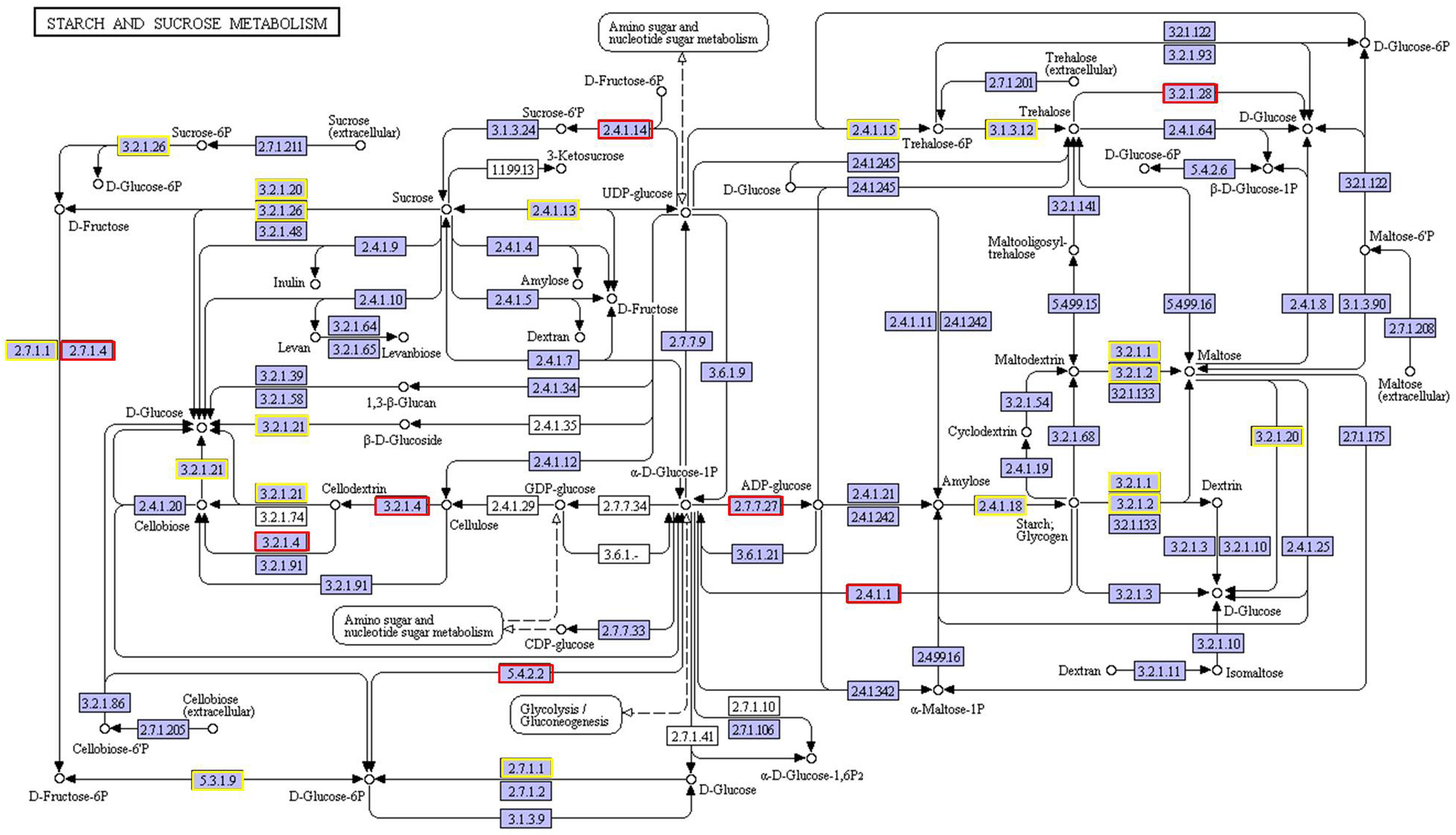

Supplement: Supplementary file 7 — Additional file 7: Figure S6. Unigenes predicted to be involved in sucrose and starch metabolism. DEGs were compared in flowers from NaCl-treated vs. control plants. Red boxes indicate significantly increased expression in NaCl-treated flowers compared to control flowers; yellow boxes indicate that some corresponding DEGs were upregulated and some were downregulated; black boxes indicate that the expression levels of the corresponding genes did not change. [file 12870_2020_2468_MOESM7_ESM.tif]
