# Supplementary material for: NaCl improves reproduction by enhancing starch accumulation in the ovules of the euhalophyte Suaeda salsa
Source: BMC Plant Biol. 2020 Jun 8;20:262. doi: 10.1186/s12870-020-02468-3 (PMC7282069; doi:10.1186/s12870-020-02468-3)
Supplement: Supplementary file 6 — Additional file 6: Table S1. DEGs annotated to photosynthesis and carbon utilization pathways in the flowers of control (0) and NaCl-treated (200 mM NaCl) S. salsa plants. [file 12870_2020_2468_MOESM6_ESM.docx]

**Additional file 6: Table S1**

Table S1 DEGs annotated to photosynthesis and carbon utilization in the flowers of *S. salsa* from control (CK) and NaCl-treated (NaCl) plants.

| Gene ID | | Control readcount | NaCl readcount | log2FC | Regulated |
| --- | --- | --- | --- | --- | --- |
| **Antena proteins** |  | |  |  |  |
| Cluster-10319.101583 | 0 | | 286.1050628 | Inf | up |
| Cluster-10319.95445 | 0 | | 174.9855128 | Inf | up |
| Cluster-10319.100112 | 0 | | 182.2574892 | Inf | up |
| Cluster-10319.94043 | 2.473532502 | | 356.3779292 | 7.1707 | up |
| **Electron transporter** |  | |  |  |  |
| Cluster-10319.101685 | 0 | | 463.5328094 | Inf | up |
| Cluster-10319.6530 | 0 | | 40.21576121 | Inf | up |
| Cluster-10319.69455 | 0 | | 24.75055314 | Inf | up |
| Cluster-10319.101701 | 0 | | 56.07763462 | Inf | up |
| Cluster-10319.6528 | 0.299214025 | | 69.28448491 | 7.8552 | up |
| Cluster-10319.104995 | 6.739395659 | | 123.1129114 | 4.1912 | up |
| **Photosynthesis** |  | |  |  |  |
| Cluster-10319.83101 | 0 | | 66.04031662 | Inf | up |
| Cluster-10319.13013 | 0 | | 49.97746473 | Inf | up |
| Cluster-10319.116023 | 0 | | 93.10544556 | Inf | up |
| Cluster-10319.134659 | 30.55469661 | | 281.759905 | 3.205 | up |
| Cluster-10319.91528 | 28.62952843 | | 715.2334079 | 4.6428 | up |
| **Carbon utilization** |  | |  |  |  |
| Cluster-10319.121478 | 0 | | 392.9568834 | Inf | up |
| Cluster-10319.71983 | 0 | | 195.807232 | Inf | up |
| Cluster-10319.9785 | 0 | | 193.4093935 | Inf | up |
| Cluster-10319.89849 | 0 | | 191.0506514 | Inf | up |
| Cluster-10319.77316 | 0 | | 126.8656012 | Inf | up |
| Cluster-10319.33950 | 0 | | 39.0319486 | Inf | up |
| Cluster-10319.34717 | 0 | | 38.25178222 | Inf | up |
| Cluster-10319.54125 | 0 | | 29.94136301 | Inf | up |
| Cluster-10319.19373 | 0 | | 27.90047195 | Inf | up |
| Cluster-10319.92536 | 0 | | 24.70009253 | Inf | up |
| Cluster-10319.133287 | 0 | | 60.06633439 | Inf | up |
| Cluster-10319.53991 | 0 | | 220.327302 | Inf | up |
| Cluster-10319.80115 | 0 | | 35.12146568 | Inf | up |
| Cluster-10319.58613 | 0 | | 39.73329467 | Inf | up |
| Cluster-10319.140916 | 0 | | 35.60588197 | Inf | up |
| Cluster-10319.96325 | 0 | | 15.01658509 | Inf | up |
| Cluster-10319.92542 | 0.850133105 | | 303.8729249 | 8.4816 | up |
| Cluster-10319.63617 | 1.165183452 | | 179.8467776 | 7.2701 | up |
| Cluster-10319.31330 | 1.700266209 | | 239.1377549 | 7.1359 | up |
| Cluster-10319.110672 | 12.81743329 | | 1214.23992 | 6.5658 | up |
| Cluster-10319.99392 | 0.283377702 | | 19.6294703 | 6.1142 | up |
| Cluster-10319.117526 | 5.08473893 | | 336.5852721 | 6.0487 | up |
| Cluster-10319.117525 | 0.299214025 | | 18.59949066 | 5.9579 | up |
| Cluster-10319.130669 | 0.897642074 | | 30.59523369 | 5.091 | up |
| Cluster-10319.117908 | 6.170740767 | | 88.07478975 | 3.8352 | up |
| Cluster-10319.86898 | 13.97101129 | | 183.1082139 | 3.7122 | up |
| Cluster-10319.182913 | 2.616698099 | | 31.83249211 | 3.6047 | up |
| Cluster-10319.79475 | 7.779564641 | | 91.09198085 | 3.5496 | up |
| Cluster-10319.99851 | 5.38417633 | | 56.25717794 | 3.3852 | up |
| Cluster-10319.146004 | 22.62055286 | | 233.2723832 | 3.3663 | up |
| Cluster-10319.106185 | 19.30745528 | | 137.6074688 | 2.8333 | up |
| Cluster-10319.110670 | 193.2132572 | | 1198.739424 | 2.6333 | up |
| Cluster-10319.130781 | 40.60738179 | | 246.1448396 | 2.5997 | up |
| Cluster-10319.119894 | 141.4394507 | | 817.8798994 | 2.5317 | up |
| Cluster-10319.110671 | 609.9078874 | | 2344.778399 | 1.9428 | up |
