# Supplementary material for: NaCl improves reproduction by enhancing starch accumulation in the ovules of the euhalophyte Suaeda salsa
Source: BMC Plant Biol. 2020 Jun 8;20:262. doi: 10.1186/s12870-020-02468-3 (PMC7282069; doi:10.1186/s12870-020-02468-3)
Supplement: Supplementary file 8 — Additional file 8: Table S2. DEGs annotated to sucrose and starch metabolism pathways in the flowers of control (0) and NaCl-treated (200 mM NaCl) plants. [file 12870_2020_2468_MOESM8_ESM.docx]

**Additional file 8: Table S2**

Table S2 DEGs annotated to sucrose and starch metabolism in the flowers of *S. salsa* from control (CK) and NaCl-treated (NaCl) plants.

| Gene ID | Control readcount | | NaCl readcount | log2FC | Regulated |
| --- | --- | --- | --- | --- | --- |
| **Sucrose metabolism** | |  |  |  |  |
| Cluster-10319.112951 | | 0 | 619.1756 | Inf | up |
| Cluster-10319.43829 | | 0 | 413.6325155 | Inf | up |
| Cluster-10319.121083 | | 0 | 454.1398239 | Inf | up |
| Cluster-10319.66232 | | 0 | 515.828613 | Inf | up |
| Cluster-10319.88385 | | 0 | 1078.659744 | Inf | up |
| Cluster-10319.145437 | | 0 | 173.1409328 | Inf | up |
| Cluster-10319.86869 | | 0 | 163.0209045 | Inf | up |
| Cluster-10319.57524 | | 0 | 133.6812475 | Inf | up |
| Cluster-10319.34971 | | 0 | 69.68793887 | Inf | up |
| Cluster-10319.63643 | | 0 | 285.7359092 | Inf | up |
| Cluster-10319.28641 | | 0 | 63.36847226 | Inf | up |
| Cluster-10319.121104 | | 0 | 63.25534682 | Inf | up |
| Cluster-10319.56420 | | 0 | 56.22653857 | Inf | up |
| Cluster-10319.104663 | | 0 | 39.44940411 | Inf | up |
| Cluster-10319.20148 | | 0 | 99.57046834 | Inf | up |
| Cluster-10319.79655 | | 0 | 32.29804725 | Inf | up |
| Cluster-10319.54267 | | 0 | 31.9103731 | Inf | up |
| Cluster-10319.51183 | | 0 | 27.45773807 | Inf | up |
| Cluster-10319.112729 | | 0 | 151.5175307 | Inf | up |
| Cluster-10319.186267 | | 0 | 26.77880203 | Inf | up |
| Cluster-10319.88276 | | 0 | 51.46551724 | Inf | up |
| Cluster-10319.74483 | | 0 | 54.00387202 | Inf | up |
| Cluster-10319.75066 | | 0 | 18.48669512 | Inf | up |
| Cluster-10319.1290 | | 0 | 18.47298195 | Inf | up |
| Cluster-10319.107039 | | 0 | 32.03676493 | Inf | up |
| Cluster-10319.51186 | | 0 | 16.71286456 | Inf | up |
| Cluster-10319.181662 | | 0 | 146.5301872 | Inf | up |
| Cluster-10319.180632 | | 0 | 15.25867635 | Inf | up |
| Cluster-10319.159616 | | 0 | 15.2242899 | Inf | up |
| Cluster-10319.105933 | | 0 | 34.29457981 | Inf | up |
| Cluster-10319.95792 | | 14.56140949 | 1373.830207 | 6.5599 | up |
| Cluster-10319.87059 | | 0.299214025 | 22.626963 | 6.2407 | up |
| Cluster-10319.105917 | | 0.299214025 | 21.55210272 | 6.1705 | up |
| Cluster-10319.56301 | | 1.009135025 | 54.12052558 | 5.745 | up |
| Cluster-10319.85241 | | 1.345513366 | 63.04904291 | 5.5502 | up |
| Cluster-10319.119329 | | 8.677206715 | 379.6156161 | 5.4512 | up |
| Cluster-10319.165807 | | 0.865969428 | 34.19988951 | 5.3035 | up |
| Cluster-10319.64804 | | 9.935731992 | 388.8631883 | 5.2905 | up |
| Cluster-10319.179089 | | 3.858109674 | 147.5699318 | 5.2574 | up |
| Cluster-10319.131820 | | 0.881805751 | 29.65310113 | 5.0716 | up |
| Cluster-10319.169757 | | 24.53219779 | 722.624314 | 4.8805 | up |
| Cluster-10319.77488 | | 2.992140247 | 70.0878232 | 4.5499 | up |
| Cluster-10319.131825 | | 2.944631277 | 56.76193762 | 4.2688 | up |
| Cluster-10319.86867 | | 3.291354271 | 60.62828698 | 4.2032 | up |
| Cluster-10319.138563 | | 7.086757342 | 128.5121819 | 4.1806 | up |
| Cluster-10319.70544 | | 7.736701857 | 137.3728657 | 4.1502 | up |
| Cluster-10319.34098 | | 1.681891708 | 28.54084843 | 4.0849 | up |
| Cluster-10319.68254 | | 5.385852444 | 83.98614961 | 3.9629 | up |
| Cluster-10319.179185 | | 88.91749628 | 1268.202834 | 3.8342 | up |
| Cluster-10319.98859 | | 8.409458541 | 118.8998897 | 3.8216 | up |
| Cluster-10319.42867 | | 5.667554031 | 77.41912865 | 3.7719 | up |
| Cluster-10319.99128 | | 30.94405901 | 422.3358678 | 3.7707 | up |
| Cluster-10319.42867 | | 5.667554031 | 77.41912865 | 3.7719 | up |
| Cluster-10319.87055 | | 4.792916065 | 53.8246586 | 3.4893 | up |
| Cluster-10319.179768 | | 3.177961831 | 35.46971631 | 3.4804 | up |
| Cluster-10319.36446 | | 13.76384513 | 136.2116821 | 3.3069 | up |
| Cluster-10319.131821 | | 5.150622401 | 49.87366039 | 3.2755 | up |
| Cluster-10319.89111 | | 49.32346209 | 464.439437 | 3.2351 | up |
| Cluster-10319.131824 | | 89.69080542 | 793.6103568 | 3.1454 | up |
| Cluster-10319.104847 | | 99.47441703 | 775.2441794 | 2.9623 | up |
| Cluster-10319.49153 | | 9.838175875 | 75.50155404 | 2.94 | up |
| Cluster-10319.111325 | | 85.75077088 | 651.4944493 | 2.9255 | up |
| Cluster-10319.57822 | | 19.53971525 | 143.3175133 | 2.8747 | up |
| Cluster-10319.57536 | | 65.89680103 | 453.2067758 | 2.7819 | up |
| Cluster-10319.101673 | | 197.7849891 | 1355.575753 | 2.7769 | up |
| Cluster-10319.28282 | | 464.8421415 | 3121.313879 | 2.7473 | up |
| Cluster-10319.106724 | | 41.8340259 | 274.7725395 | 2.7155 | up |
| Cluster-10319.92468 | | 193.6443158 | 1208.278757 | 2.6415 | up |
| Cluster-10319.87051 | | 75.96050099 | 447.2770471 | 2.5578 | up |
| Cluster-10319.87056 | | 134.6772194 | 755.945998 | 2.4888 | up |
| Cluster-10319.84480 | | 30.3122822 | 168.9519351 | 2.4786 | up |
| Cluster-10319.106725 | | 725.2021792 | 4017.374853 | 2.4698 | up |
| Cluster-10319.87053 | | 589.8527475 | 3157.709257 | 2.4205 | up |
| Cluster-10319.187122 | | 24.46588415 | 123.072081 | 2.3307 | up |
| Cluster-10319.105916 | | 68.64442058 | 325.8512079 | 2.247 | up |
| Cluster-10319.66220 | | 174.9500413 | 783.5607008 | 2.1631 | up |
| Cluster-10319.105934 | | 87.71621565 | 372.8386889 | 2.0876 | up |
| **Starch metabolism** | |  |  |  |  |
| Cluster-10319.64215 | | 0 | 603.1038146 | Inf | up |
| Cluster-10319.100482 | | 0 | 311.8375239 | Inf | up |
| Cluster-10319.107882 | | 0 | 278.1820158 | Inf | up |
| Cluster-10319.10074 | | 0 | 208.4798445 | Inf | up |
| Cluster-10319.90419 | | 0 | 372.8158605 | Inf | up |
| Cluster-10319.95740 | | 0 | 161.0528753 | Inf | up |
| Cluster-10319.179563 | | 0 | 116.7123655 | Inf | up |
| Cluster-10319.156265 | | 0 | 263.2805534 | Inf | up |
| Cluster-10319.36803 | | 0 | 170.7165763 | Inf | up |
| Cluster-10319.153318 | | 0 | 63.96599919 | Inf | up |
| Cluster-10319.185825 | | 0 | 52.24064657 | Inf | up |
| Cluster-10319.112891 | | 0 | 45.82038158 | Inf | up |
| Cluster-10319.119025 | | 0 | 37.61815283 | Inf | up |
| Cluster-10319.140235 | | 0 | 60.70425972 | Inf | up |
| Cluster-10319.112216 | | 0 | 28.78015269 | Inf | up |
| Cluster-10319.168626 | | 0 | 28.77962458 | Inf | up |
| Cluster-10319.66922 | | 0 | 27.38982317 | Inf | up |
| Cluster-10319.6437 | | 0 | 27.00718012 | Inf | up |
| Cluster-10319.116554 | | 0 | 108.8900284 | Inf | up |
| Cluster-10319.172382 | | 0 | 23.58431172 | Inf | up |
| Cluster-10319.9922 | | 0 | 22.10741914 | Inf | up |
| Cluster-10319.179240 | | 0 | 20.56175366 | Inf | up |
| Cluster-10319.90313 | | 0 | 20.41285567 | Inf | up |
| Cluster-1476.0 | | 0 | 19.40698131 | Inf | up |
| Cluster-10319.122819 | | 0 | 33.74751944 | Inf | up |
| Cluster-10319.9670 | | 0 | 17.48478086 | Inf | up |
| Cluster-10319.132940 | | 0 | 20.30561933 | Inf | up |
| Cluster-10319.96821 | | 0.850133105 | 242.2977686 | 8.1549 | up |
| Cluster-10319.113954 | | 1.837940136 | 335.6399206 | 7.5127 | up |
| Cluster-10319.100276 | | 6.011738847 | 460.6520692 | 6.2598 | up |
| Cluster-10319.63672 | | 7.274478416 | 428.946608 | 5.8818 | up |
| Cluster-10319.4509 | | 1.009135025 | 41.92799795 | 5.3767 | up |
| Cluster-10319.152912 | | 1.681891708 | 52.41449303 | 4.9618 | up |
| Cluster-10319.25388 | | 6.409562992 | 177.3607135 | 4.7903 | up |
| Cluster-10319.155177 | | 7.911124786 | 218.1808329 | 4.7855 | up |
| Cluster-10319.5864 | | 26.57388899 | 578.6596778 | 4.4446 | up |
| Cluster-10319.66470 | | 28.14428775 | 395.6631984 | 3.8134 | up |
| Cluster-10319.110464 | | 13.85718696 | 187.0966799 | 3.7551 | up |
| Cluster-10319.23213 | | 66.22092382 | 781.4267779 | 3.5608 | up |
| Cluster-10319.47468 | | 13.8324753 | 151.3901432 | 3.4521 | up |
| Cluster-10319.51148 | | 12.10773567 | 116.8994932 | 3.2713 | up |
| Cluster-10319.125532 | | 18.51539917 | 174.8564906 | 3.2394 | up |
| Cluster-10319.185213 | | 15.05236694 | 127.1353259 | 3.0783 | up |
| Cluster-10319.27670 | | 19.7101324 | 151.9500528 | 2.9466 | up |
| Cluster-10319.142360 | | 58.17129276 | 447.9322433 | 2.9449 | up |
| Cluster-10319.23209 | | 145.792034 | 1099.414731 | 2.9148 | up |
| Cluster-10319.54048 | | 20.04733965 | 139.0139745 | 2.7937 | up |
| Cluster-10319.75706 | | 10.39880092 | 70.85640364 | 2.7685 | up |
| Cluster-10319.98633 | | 131.6753252 | 887.4684969 | 2.7527 | up |
| Cluster-10319.97679 | | 225.5956418 | 1387.829222 | 2.621 | up |
| Cluster-10319.161445 | | 166.0317575 | 915.2545565 | 2.4627 | up |
| Cluster-10319.44186 | | 69.31655516 | 358.9341702 | 2.3724 | up |
| Cluster-10319.166081 | | 90.45749805 | 465.7135123 | 2.3641 | up |
| Cluster-10319.80580 | | 1216.016066 | 6037.139345 | 2.3117 | up |
| Cluster-10319.40263 | | 118.196198 | 548.0876749 | 2.2132 | up |
