# Supplementary material for: NaCl improves reproduction by enhancing starch accumulation in the ovules of the euhalophyte Suaeda salsa
Source: BMC Plant Biol. 2020 Jun 8;20:262. doi: 10.1186/s12870-020-02468-3 (PMC7282069; doi:10.1186/s12870-020-02468-3)
Supplement: Supplementary file 9 — Additional file 9: Table S3. Primer pairs used for quantitative RT-PCR. [file 12870_2020_2468_MOESM9_ESM.docx]

**Additional file 9: Table S3**

Table S3 Primer pairs for real-time quantitative PCR

| Genes | Sense primer ( 5’- 3’) | Antisense primer ( 5’- 3’) |
| --- | --- | --- |
| *Actine* | GCTCTACCCCATGCAATCCT | TGCTCTTGGCAGTCTCTGATT |
| *Ss160205* | TGCCCTTGTGGACAAGAT | AAGGCTGCACATCCAGGCTCA |
| *Ss91528* | ATGATGAAGATGATGATGAT | TGAACTCCAGACATATAGCA |
| *Ss80498* | TTAATGTGTCCACCAGAA | GAATGCTGCTGATGATTG |
| *Ss79658* | ATTCAAGCCACCAATTACC | TTCACCAACATCTTCATACAA |
| *Ss94819* | CAGGTAATAATAGCAGCACTAACA | GATGATTGATGATGGATGATGGA |
| *Ss73638* | GTGGTGGTGTTGTTGTTG | AACCTTGGCATACCTCAC |
| *Ss38466* | AGCAGGGTTATAGAAGGT | TAAGTAATGGAAGATACAAGAGT |
| *Ss182028* | TCAGGATGCATCGCCACACA | TGTCATGCCCGAGGAGCG |
| *Ss23889* | CATAAGTAATTTGTATAACTAAT | ACCTGGATAACATTCAGAATAACT |
| *Ss71871* | ATAGCGGTTCTTGAACCACAGTTA | CATCACATTGAGATCTCCAGCATG |
